# microRNA-132 attenuates inflammation in induced pluripotent stem cell-derived microglia from Alzheimer’s disease patients

**DOI:** 10.1186/s40478-026-02228-8

**Published:** 2026-03-07

**Authors:** Amber Penning, Sarah Snoeck, Olmo Ruiz Ormaechea, Dilara Ayyildiz, Oliver Polzer, Martin Buitrago-Arango, Raffaella Capobianco, Fred de Winter, Sriram Balusu, Joost Verhaagen, Carlos P. Fitzsimons, Constantin d’Ydewalle, Paul J. Lucassen, Dieder Moechars, Lujia Zhou, Evgenia Salta

**Affiliations:** 1 Netherlands Institute for Neuroscience, Amsterdam, The Netherlands; 2 Brain Plasticity Group, Swammerdam Institute for Life Sciences, Faculty of Science, University of Amsterdam, Amsterdam, The Netherlands; 3 VIB-KU Leuven Center for Brain & Disease Research, Leuven, Belgium; 4 Neuroscience Discovery, Janssen Research & Development, Janssen Pharmaceutica NV, Beerse, Belgium

**Keywords:** microRNA, miR-132, iPSC, Microglia, Alzheimer’s disease

## Abstract

Microglia, the resident immune cells of the brain, are increasingly recognized as key contributors to Alzheimer’s disease (AD) pathology. Multiple studies have identified microRNA-132 (miR-132) as one of the most significantly downregulated microRNAs in AD. Apart from well-established pleiotropic regulatory functions in neurons, previous evidence also suggested a role for miR-132 in regulating (neuro)inflammation. Yet, the precise mechanisms by which miR-132 impacts microglia remain unknown. In this study, we investigated the role of miR-132 in modulating microglial gene expression and function using gain- and loss-of-function approaches in human-induced pluripotent stem cell (iPSC)-derived microglia (iMGs) from both healthy controls and sporadic AD (sAD) patients. Our findings indicate that while miR-132 may not be indispensable for some baseline microglial functions, increasing its expression in sAD iMGs can reverse disease-associated gene expression changes and attenuate inflammatory responses. To further explore its therapeutic potential, we overexpressed miR-132 in hippocampal neurons of an AD mouse model, employing a clinically relevant adeno-associated viral (AAV) delivery method. miR-132 overexpression was well-tolerated and induced non-cell autonomous effects in microglia. This study sheds light into the regulatory role of miR-132 in microglia under both physiological and AD conditions, and emphasizes the importance of optimizing safe dosage parameters for future clinical applications.

**Supplementary Information:**

The online version contains supplementary material available at 10.1186/s40478-026-02228-8.

## Introduction

Alzheimer’s disease (AD) is a neurodegenerative disorder characterized by progressive memory loss and cognitive impairment, thought to be caused by complex dyshomeostasis in the brain [1]. The current scarcity of effective treatments for AD may -at least partially- reflect the absence of a single, main pathogenic mechanism that leads to the molecular and cellular alterations occurring in the AD brain [1].

Substantial recent evidence has demonstrated that microRNAs (miRNAs) are dysregulated already early on in AD pathology [2–7]. miRNAs are small non-coding RNAs that regulate gene expression post-transcriptionally by either blocking translation or inducing mRNA degradation of their target mRNA transcripts [8–10]. Since target binding does not require full complementarity, miRNAs can simultaneously regulate multiple target genes, generating intricate molecular networks, in which both mRNAs and other miRNAs become involved in various cellular processes and pathways [8, 11, 12]. This pleiotropic nature and broad functionality confer miRNAs the potential to regulate multiple key checkpoints within disease pathways, and position them as promising therapeutic candidates, especially for complex, multigenic and multifactorial diseases, like AD [13–17].

One compelling example of a miRNA with potential therapeutic relevance in AD is microRNA-132 (miR-132), the most consistently and robustly downregulated miRNA in human AD brain [2, 18, 19]. miR-132 can ameliorate AD pathology at multiple levels, through its involvement in TAU metabolism and hyperphosphorylation, amyloidosis, neuronal survival, adult neurogenesis and memory formation [18, 20–34].

While amyloid-β plaques and TAU tangles have long been recognized as pathological hallmarks of AD, emerging research highlights the critical role of neuroinflammation in disease progression [1, 35–37]. Microglia, the brain-resident immune cells, play a pivotal role in orchestrating inflammatory responses in the central nervous system [38]. Dysfunctional microglial activation has been implicated in AD, contributing to synaptic loss, neuronal damage, and chronic inflammation [1, 38, 39]. miR-132, classified as a ‘NeurimmiR’, operates at the interface of both immune and neuronal functions [18, 40, 41]. Recent findings suggest that miR-132 can regulate microglial homeostasis and induce transition across different activation states under physiological conditions [42]. However, the exact regulatory mechanisms by which miR-132 affects microglial function in AD remain unknown.

In this study, we systematically profiled the biological pathways via which miR-132 regulation converges in microglia, identified putative miR-132 targets mediating these effects and assessed the impact on microglial function, using human induced pluripotent stem cell (iPSC)-derived microglia-like cells (iMGs). We further establish that iMGs derived from sAD patients exhibit similarities to microglia in AD brain. Additionally, we show that miR-132 exerts anti-inflammatory effects in sAD iMGs. Complementary to this observation, in vivo neuronal miR-132 overexpression in AD mice induces non-cell-autonomous effects in microglia, putatively altering their activation profile and the interaction with amyloid-β plaques. Our findings elucidate the regulatory role of miR-132 in microglia under physiological and AD conditions, thereby contributing to a better understanding and more precise evaluation of its therapeutic potential in AD.

## Results

### miR-132 loss-of-function in healthy control iMGs alters microglial gene expression, without affecting their functional output

**Fig. 1:**
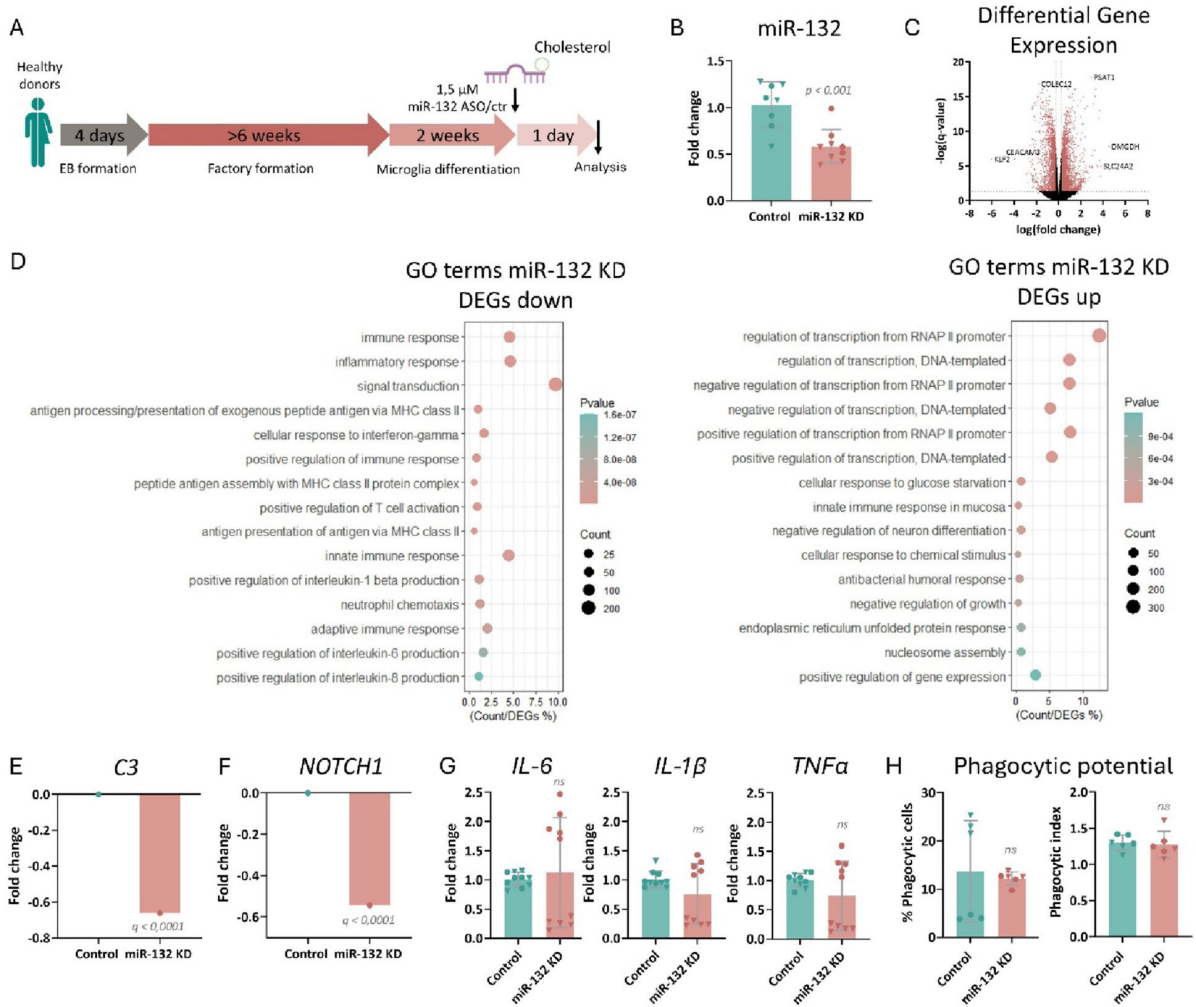
Impact of miR-132 loss-of-function in healthy control iMGs. **A** Schematic representation of the experimental procedure employed for differentiating iPSC-derived microglia and knocking down miR-132. **B** Semi-quantitative real-time PCR of miR-132 levels upon miR-132 knockdown in mature iMGs. n = 4–5 technical replicates per iPSC line; 2 iPSC lines in total. Student’s t-test was applied for statistical analysis. **C** Volcano plot depicting all genes identified by RNA sequencing, with significant differentially expressed genes with fold change > 0.25 (Upregulated) or < − 0.25 (Downregulated) shown in pink. n = 5 technical replicates per iPSC line; 2 iPSC lines in total. **D** GO term enrichment analysis. Top fifteen significantly enriched pathways are shown. A full list can be found in Supplementary Table 3. **E**, **F** Differential expression analysis between miR-132 knockdown and control group for *C3* (**E**) and *NOTCH1* (**F**). **G** Semi-quantitative real-time PCR of *IL-6*, *IL-1β* and *TNF-α* levels after LPS stimulation upon miR-132 KD in mature iMGs. n = 5 technical replicates per iPSC line; 2 iPSC lines in total. Mann–Whitney U Test was applied for statistical analysis. **H** Quantification of the percentage of iMGs that engulfed fluorescent beads compared to the total quantified iMG number, and the phagocytic index, calculated as the average number of engulfed beads per phagocytic cell. n = 3 technical replicates per iPSC line; 2 iPSC lines in total. Student’s t-test was applied for statistical analysis. Data point symbols indicate the origin of the iPSC lines.

To investigate whether miR-132-dependent regulation of gene expression is required in microglia, we used a loss-of-function approach in iMGs derived from healthy control donors (Fig. 1A) (information on iPSC lines in Supplementary Table 1). Upon iPSC differentiation, a microglia-like phenotype was confirmed with both immunolabeling and gene expression profiling using markers of mature microglia (IBA1, TMEM119, *HEXB*, *PROS1*, *MERTK*) (Supplementary Fig. 1A, B). Additionally, the absence of non-microglial cell types was confirmed by assessing the expression levels of a panel of neuronal, astrocytic and oligodendrocytic marker genes directly in our cultures (Supplementary Fig. 1C, D). To induce miR-132 knockdown (KD), we treated iMGs with a cholesterol-conjugated miR-132 antisense or control oligonucleotide for 24 h (Fig. 1B) and assessed the effects on the transcriptome using bulk RNA sequencing. The duration of treatment was optimized by confirming the effect of miR-132 KD on a previously identified microglial miR-132 target, *CD164* [42] (Supplementary Fig. 2A). Differential gene expression analysis between miR-132 KD and control iMGs yielded a total of 4545 significant differentially expressed genes (DEGs) (Fig. 1C, Supplementary Table 2).

To further characterize the effects of miR-132 loss-of-function in iMGs, pathway enrichment analysis was performed among either downregulated or upregulated DEGs using DAVID. Gene ontology (GO) (biological process) analysis revealed enrichment of multiple immune and inflammatory response pathways among downregulated DEGs, indicative of the involvement of miR-132 in the inflammatory response (Fig. 1D). This was further exemplified by the significant downregulation of complement *C3*, a major component of the complement system, and key player in the immune response [43] (Fig. 1E). Additionally, miR-132 KD induced enrichment of pathways involved in neuronal differentiation and neural stem cell maintenance among the upregulated DEGs, suggesting a potential role for miR-132 in microglia-neuron crosstalk in neurogenesis (Fig. 1D, Supplementary Table 3).

Previous work has shown that miR-132 is a potent regulator of Notch1 signaling in radial glial cells, i.e. neural stem cells [44]. We confirmed this regulatory role of miR-132 in iMGs as well, where miR-132 loss-of-function significantly downregulated *NOTCH1* (Fig. 1F).

We then asked whether the miR-132 deficiency-induced transcriptomic alterations translate into effects on microglial function, focusing on two of the main physiological roles of microglia, namely inflammatory response and phagocytosis. We first confirmed that an inflammatory response was elicited following application of lipopolysaccharide (LPS) to healthy control iMGs (Supplementary Fig. 1F). LPS was employed as a well-characterized inflammatory stimulus that reliably and consistently induces the microglial activation axis (pro-inflammatory cytokines, transcriptional reprogramming, changes in phagocytic state) across iPSC-derived preparations and towards AD-relevant profiles [45–49]. Interestingly, treatment with LPS also induced an increase in miR-132 levels per se (Supplementary Fig. 1G), as shown before in astrocytes and murine microglia [41, 50]. We next monitored the effects of LPS treatment after miR-132 KD (Supplementary Fig. 3A). We did not observe any significant changes in the expression levels of inflammatory markers upon miR-132 KD (Fig. 1G). Similarly, upon treatment with fluorescent beads to monitor phagocytosis (Supplementary Fig. 2F), miR-132 depletion did not influence the percentage of phagocytic cells or the phagocytic index, which reflects the average number of engulfed beads per phagocytic cell (Fig. 1H).

Together, these data suggest that even though miR-132 loss-of-function in healthy control iMGs does have an effect on gene expression programs involving pathways related to both inflammation and neurogenesis, this does not result in a detectable effect in their inflammatory response or phagocytic activity, at least under the experimental conditions tested here.

### iMGs from sAD patients exhibit transcriptional and functional similarities to AD microglia

**Fig. 2:**
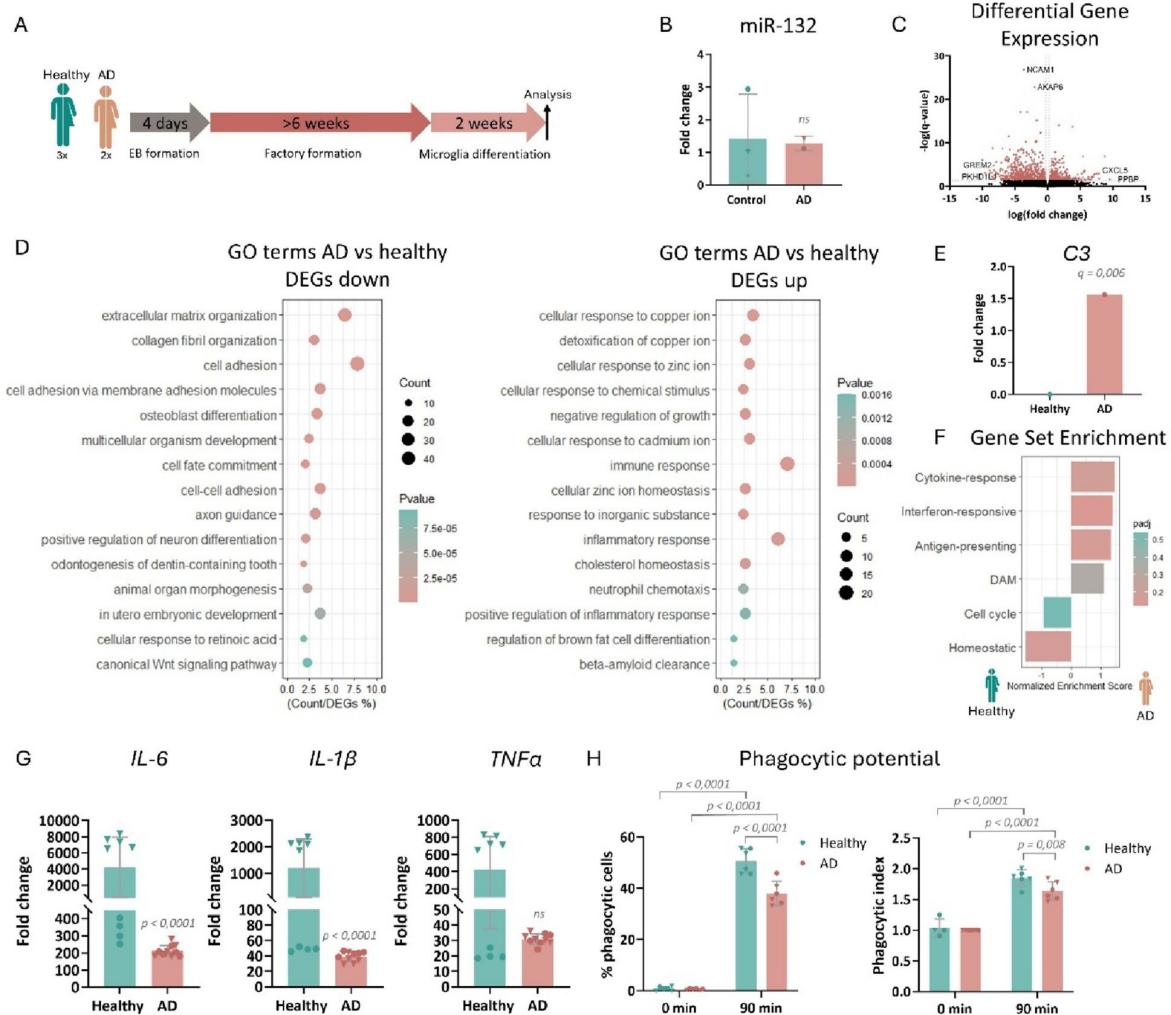
Comparison between healthy control and sAD iMGs. **A** Schematic representation of the experimental procedure employed for comparing iPSC-derived microglia from healthy controls and sAD patients. **B** Semi-quantitative real-time PCR of miR-132 in mature iMGs. n = 3 healthy control iPSC lines and 2 sAD iPSC lines. **C** Volcano plot depicting all genes identified by RNA sequencing, with significant differentially expressed genes with fold change > 0.25 (Upregulated) or < -0.25 (Downregulated) shown in pink. n = 5 technical replicates per iPSC line; 3 healthy control iPSC lines and 2 sAD iPSC lines in total. **D** GO term enrichment analysis. Top fifteen significantly enriched pathways are shown. A full list can be found in Supplementary Table 3. **E** Differential expression analysis between healthy control and sAD iMGs for complement factor *C3*. **F** Gene set enrichment analysis ran for microglial signature gene sets (Supplementary Table 4) **G** Semi-quantitative real-time PCR of *IL-6*, *IL-1β* and *TNF-α* levels after LPS stimulation in mature iMGs. n = 5 technical replicates per iPSC line; 2 healthy control iPSC lines and 2 sAD iPSC lines in total. Mann–Whitney U Test was applied for statistical analysis. **H** Quantification of the percentage of iMGs that engulfed fluorescent beads compared to the total quantified iMG number, and the phagocytic index, calculated as the average number of engulfed beads per phagocytic cell. n = 3 technical replicates per iPSC line; 2 healthy control iPSC lines and 2 sAD iPSC lines in total. Two-way ANOVA with Tukey’s correction was used for statistical analysis. Data point symbols indicate the origin of the iPSC lines.

To assess whether iMGs derived from sAD patients recapitulate AD endophenotypes, we first compared healthy control and sAD iMGs based on their transcriptional and functional profiles (Fig. 2A, Supplementary Fig. 2C, D). Gene expression levels of microglial, neuronal, astrocytic and oligodendrocytic markers confirmed once again the microglial identity of our cultures (Supplementary Fig. 1E). No difference in miR-132 levels was observed (Fig. 2B). Transcriptional profiling via bulk RNA sequencing identified a total of 833 DEGs between healthy and sAD iMGs (Fig. 2C, Supplementary Table 2).

Pathway enrichment analysis revealed that in sAD iMGs (compared to iMGs from healthy control donors) pathways related to neuronal function were enriched among downregulated DEGs, which could be suggestive of potentially altered intercellular signaling from microglia towards neurons (Fig. 2D). Among upregulated DEGs, sAD iMGs showed enrichment of both immune and inflammatory response pathways, confirming previous findings [51] (Fig. 2D, Supplementary Table 3). Interestingly, sAD iMGs additionally showed upregulation of complement component *C3* (Fig. 2E), which is also elevated in the brain of AD patients and required for neurodegeneration [52].

To assess differences in microglial cell states, we performed a meta-analysis of previously published single-cell and single-nucleus RNA sequencing datasets from microglia isolated from postmortem human AD brain and human microglia transplanted into AD mouse brain [53–57] to obtain a list of markers for microglial cell state signatures found in AD (Supplementary Table 4). Gene set enrichment analysis [58, 59] (GSEA) using this list confirmed a non-significant trend for enrichment of inflammatory microglial signatures in sAD iMGs, whereas a homeostatic microglial signature was enriched in healthy iMGs (Fig. 2F).

Next, we investigated whether sAD iMGs model AD-like phenotypes on a functional level. Similarly to healthy control iMGs, treatment with LPS induced an increase in miR-132 levels in sAD iMGs (Supplementary Fig. 1G). Even though both groups were able to elicit an inflammatory response (Supplementary Fig. 1F), the induction of the inflammatory genes *IL-6* and *IL-1β* was lower in sAD iMGs after LPS stimulation compared to healthy control iMGs. No difference in the induction of *TNF-α* was detected (Fig. 2G). This dampened inflammatory response in sAD iMGs may suggest that even though baseline levels of inflammatory genes are higher, sAD iMGs are less capable of responding to inflammatory triggers. Similarly, although both groups could perform phagocytosis, both the percentage of phagocytic cells and the phagocytic index were lower in AD than in healthy control iMGs (Fig. 2H, Supplementary Fig. 2F), as has been previously reported in human iPSC-derived microglia with altered expression of AD risk genes [60–64].

Collectively, our data indicate that iMGs from AD patients recapitulate distinct transcriptional and functional endophenotypes of human AD microglia and can, thus, be used to model certain aspects of AD pathology in vitro.

### miR-132 reverses pathways altered in sAD iMGs

**Fig. 3:**
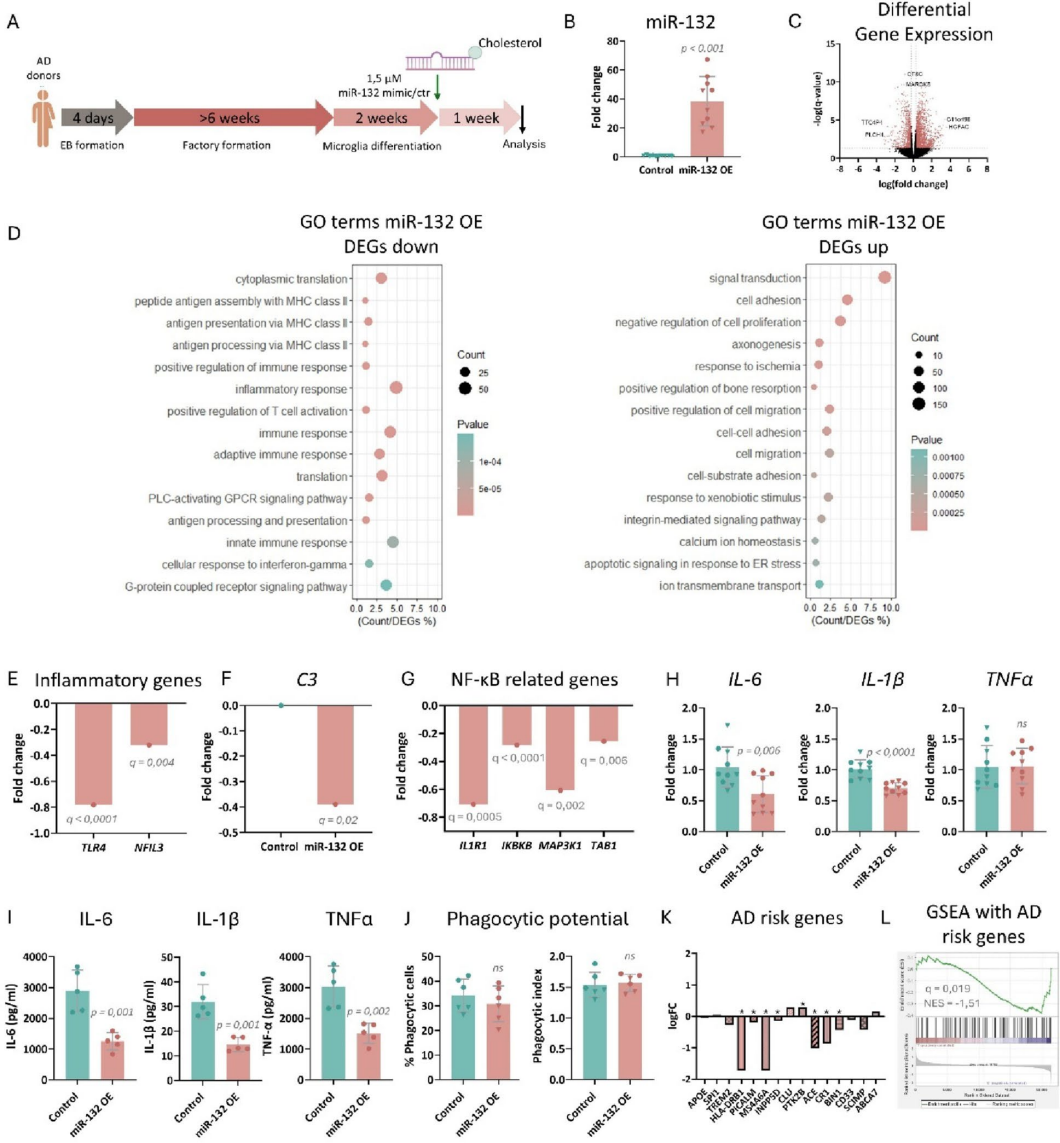
Impact of miR-132 overexpression in sAD iMGs. **A** Schematic representation of the experimental procedure employed for differentiating iPSC-derived microglia and overexpressing miR-132. **B** Semi-quantitative real-time PCR of miR-132 levels upon miR-132 overexpression in mature iMGs. n = 5 technical replicates per iPSC line; 2 iPSC lines in total. Student’s t-test was applied for statistical analysis. **C** Volcano plot depicting all genes identified by RNA sequencing, with significant differentially expressed genes with fold change > 0.25 (Upregulated) or < -0.25 (Downregulated) shown in pink. n = 5 technical replicates per iPSC line; 2 iPSC lines in total. **D** GO term enrichment analysis. Top fifteen significantly enriched pathways are shown. A full list can be found in Supplementary Table 3. **E**, **F** Differential expression analysis between miR-132 overexpression and control group for *TLR4, NFIL3* (**E**) and *C3* (**F**). **G** Differential expression analysis between miR-132 overexpression and control group for *IL1R1*, *IKBKB*, *MAP3K1* and *TAB1* (BIOCARTA_NFKB_PATHWAY). **H** Semi-quantitative real-time PCR of *IL-6*, *IL-1β* and *TNF-α* levels after LPS stimulation upon miR-132 overexpression in mature iMGs. n = 5 technical replicates per iPSC line; 2 iPSC lines in total. Student’s t-test was applied for statistical analysis. **I** Levels of secreted cytokines IL-6, IL-1β and TNF-α, measured in conditioned medium of LPS-stimulated AD iMGs upon miR-132 overexpression. n = 5 technical replicates. Student’s t-test was applied for statistical analysis. **J** Quantification of the percentage of iMGs that engulfed fluorescent beads compared to the total quantified iMG number, and the phagocytic index, calculated as the average number of engulfed beads per phagocytic cell. n = 3 technical replicates per iPSC line; 2 iPSC lines in total. Student’s t-test was applied for statistical analysis. **K** Differential expression analysis between miR-132 overexpression and control group for the 15 AD risk genes with highest expression in our dataset. **L** Gene Set Enrichment Analysis (GSEA) ran for AD risk genes (Supplementary Table 4). Data point symbols indicate the origin of the iPSC lines.

To study whether miR-132 supplementation could reverse any of the phenotypes we identified in sAD iMGs, cells were treated with a cholesterol-conjugated miR-132 mimic or control oligonucleotide, resulting in robust miR-132 increase (Fig. 3A, B). The duration of treatment was based on previously published literature [42]. We additionally observed a significant downregulation of *CD164*, a previously characterized miR-132 target [42], thus confirming functional miR-132 regulation (Supplementary Fig. 2B). Bulk RNA sequencing revealed 2666 DEGs between the miR-132 and control group (Fig. 3C, Supplementary Table 2).

miR-132 supplementation in sAD iMGs led to downregulation of genes involved in immune response (Fig. 3D), whereas immune response and inflammatory pathways were previously enriched among upregulated DEGs in sAD iMGs when compared to healthy control cells (Fig. 2D). Conversely, gene signatures related to neuritogenesis were enhanced among the upregulated DEGs (Fig. 3D), as opposed to what we observed in AD versus healthy control iMGs (Fig. 2D, Supplementary Table 3). The suppression of pro-inflammatory genes was exemplified by the significant downregulation of both *TLR4,* a key activator of the immune response [65], and *NFIL3*, which is an upstream regulator of NF-κB-mediated inflammation [66] (Fig. 3E). Interestingly, miR-132 overexpression also significantly downregulated complement component *C3* (Fig. 3F), which was increased in AD compared to healthy control iMGs (Fig. 2E). To further assess the effect of miR-132 on the inflammatory response we monitored changes in the genes involved in the NF-κB pathway, a key mediator of inflammation. We identified four genes significantly changing, all of which were significantly downregulated upon miR-132 supplementation (Fig. 3G), further suggesting that miR-132 suppresses pro-inflammatory genes and pathways.

We next compared the enrichment of a comprehensive list of reference microglial cell state transcriptional signatures (Supplementary Table 4) upon miR-132 supplementation in AD iMGs to those altered in AD versus healthy iMGs. miR-132 significantly enhanced signatures related to microglial activation and phagocytosis, whereas it attenuated pro-inflammatory cell state signatures that were significantly enriched in AD, like those corresponding to disease-associated (DAM), inflammatory, interferon-responsive, antigen-presenting, cytokine-responsive, and senescence-associated microglia (Supplementary Fig. 3D). Ingenuity Pathway Analysis confirmed that miR-132 supplementation in AD iMGs reversed signatures related to cellular functions that were either enhanced or repressed in AD compared to healthy cells. More specifically, miR-132 supplementation activated cell migration, survival and proliferation, while it inhibited apoptosis, necrosis and inflammatory response (Supplementary Fig. 3E). FOXA1, a predicted miR-132 target whose repression by miR-132 has been reported to ameliorate cognition in AD rats [67], was predicted to be an activated upstream regulator among the genes differentially expressed between AD and healthy iMGs (Supplementary Fig. 3F); reversely, FOXA1 was predicted to become deactivated upon miR-132 supplementation in AD iMGs (Supplementary Fig. 3F).

To probe the impact of increasing miR-132 levels on cellular function, we next stimulated sAD iMGs with LPS after miR-132 supplementation (Supplementary Fig. 3B). We observed a partially dampened inflammatory response, wherein the levels of *IL-6* and *IL-1*β significantly decreased compared to the control treatment group (Fig. 3H). Interestingly, the secreted levels of IL-6, IL-1β and TNFα, significantly decreased after LPS treatment when comparing miR-132- to the control oligonucleotide-treated group (Fig. 3I, Supplementary Fig. 3G). However, miR-132 supplementation in sAD iMGs, did not impact the phagocytotic potential of the cells (Fig. 3J).

Finally, we asked whether miR-132 could regulate gene signatures related to AD risk in sAD iMGs. We assessed the levels of a list of genome-wide association studies (GWAS)-significant gene candidates, extracted from the most recent meta-analysis of genetic AD datasets [53] (Supplementary Table 4). Interestingly, increasing miR-132 levels significantly suppressed the majority of the 15 AD risk genes with the highest expression in our dataset (Fig. 3K), and GSEA including the full list of AD risk genes revealed an enrichment in the control-treated compared to the miR-132-treated cells (Fig. 3L).

Our findings indicate that increasing miR-132 in sAD iMGs can reverse several gene expression alterations seen in AD compared to healthy control iMGs and partially dampen their inflammatory response.

### miR-132 supplementation in healthy control iMGs

**Fig. 4:**
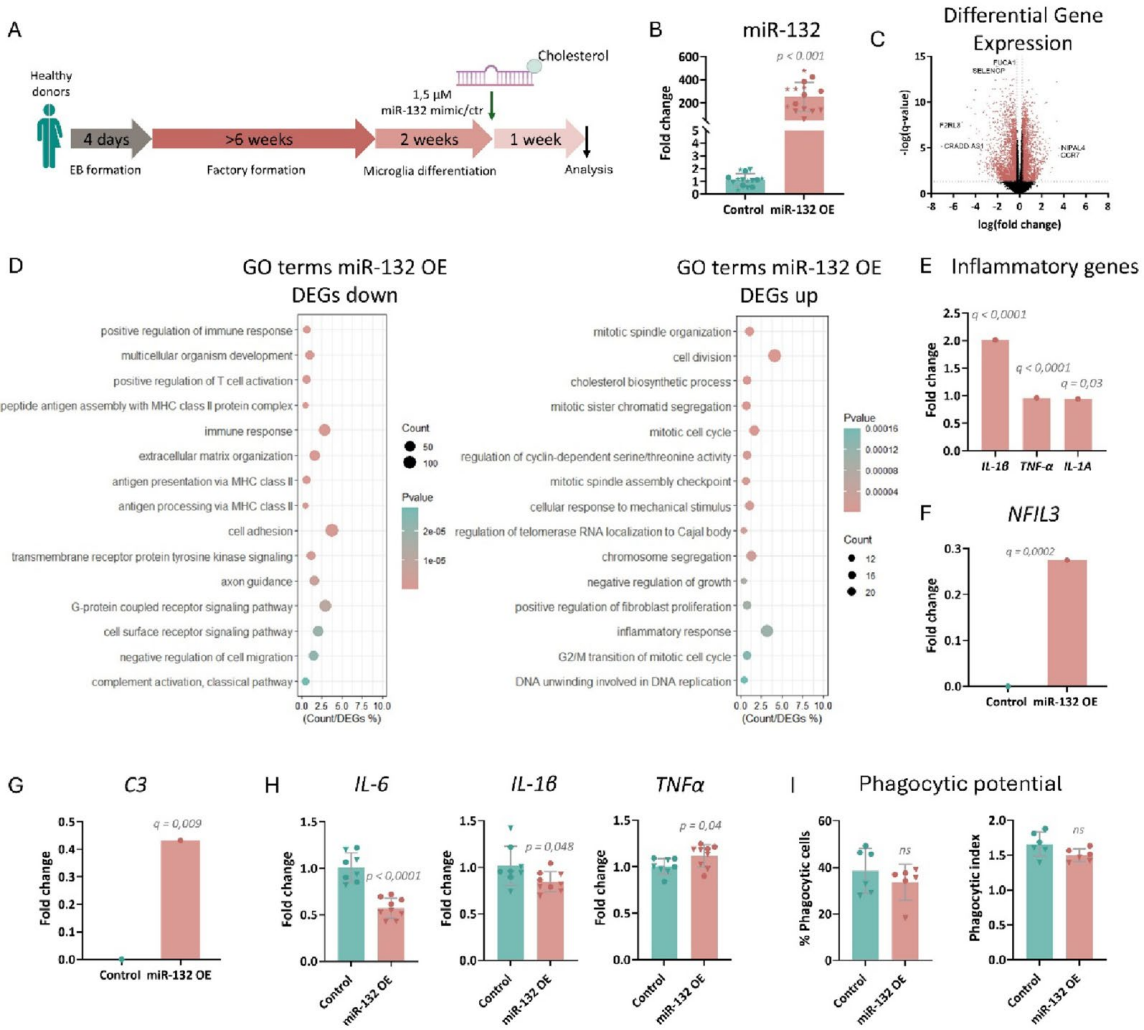
Impact of miR-132 overexpression in healthy control iMGs. **A** Schematic representation of the experimental procedure employed for differentiating iPSC-derived microglia and overexpressing miR-132. **B** Semi-quantitative real-time PCR of miR-132 levels upon miR-132 overexpression in mature iMGs. n = 4–5 technical replicates per iPSC line; 3 iPSC lines in total. Student’s t-test was applied for statistical analysis. **C** Volcano plot depicting all genes identified by RNA sequencing, with significant differentially expressed genes with fold change > 0.25 (Upregulated) or < − 0.25 (Downregulated) shown in pink. n = 5 technical replicates per iPSC line; 3 iPSC lines in total. **D** GO term enrichment analysis. Top fifteen significantly enriched pathways are shown. A full list can be found in Supplementary Table 3. **E**–**G** Differential expression analysis between miR-132 overexpression and control group for *IL-1β, TNF-α, IL-1A* (**E**), *NFIL3* (**F**) and *C3* (**G**). **H** Semi-quantitative real-time PCR of *IL-6*, *IL-1β* and *TNF-α* levels after LPS stimulation upon miR-132 KD in mature iMGs. n = 5 technical replicates per iPSC line; 2 iPSC lines in total. Student’s t-test was applied for statistical analysis. **I** Quantification of the percentage of iMGs that engulfed fluorescent beads compared to the total quantified iMG number, and the phagocytic index, calculated as the average number of engulfed beads per phagocytic cell. n = 3 technical replicates per iPSC line; 2 iPSC lines in total. Student’s t-test was applied for statistical analysis. Data point symbols indicate the origin of the iPSC lines.

Increasing miR-132 levels beyond a certain context-dependent dose threshold may induce cellular toxicity that could eventually also impact functional output, as we have previously observed in mouse hippocampus [31]. To explore whether enhancing miR-132 levels beyond baseline could induce aberrant gene expression, we treated healthy control iMGs with a miR-132 or control oligonucleotide (Fig. 4A, B), and identified 4991 DEGs between the miR-132 supplementation and control group upon genome-wide transcriptomic profiling using bulk RNA sequencing as before (Fig. 4C, Supplementary Table 2).

miR-132 overexpression led to the enrichment of many pathways involved in cell cycle when analysing GO biological processes among upregulated DEGs (Fig. 4D), similar to what has been previously reported [31]. Interestingly, immune response pathways were enriched among downregulated DEGs, while the inflammatory response was enriched among upregulated DEGs. (Fig. 4D, Supplementary Table 3). This altered inflammatory status was exemplified by a significant upregulation of the pro-inflammatory genes *IL-1β*, *TNF-α, IL-1A* (Fig. 4E) and *NFIL3* (Fig. 4F). Opposite to the effect of miR-132 loss-of-function in healthy control iMGs that led to decreased *C3* levels (Fig. 1E), miR-132 supplementation in healthy control iMGs increased *C3* expression (Fig. 4G).

Increasing miR-132 (Supplementary Fig. 3C) prior to inducing the inflammatory response with LPS resulted in the downregulation of *IL-6* and *IL-1β*, whereas *TNF-α* was upregulated (Fig. 4H). Like before, no effect was observed in the phagocytic potential of the miR-132-treated healthy control iMGs (Fig. 4I).

Taken together, increasing miR-132 levels in healthy control iMGs has a bimodal effect on inflammation, reflected by both increased and decreased levels of inflammatory markers.

### miR-132 prioritizes its targetome depending on the cellular context

**Fig. 5:**
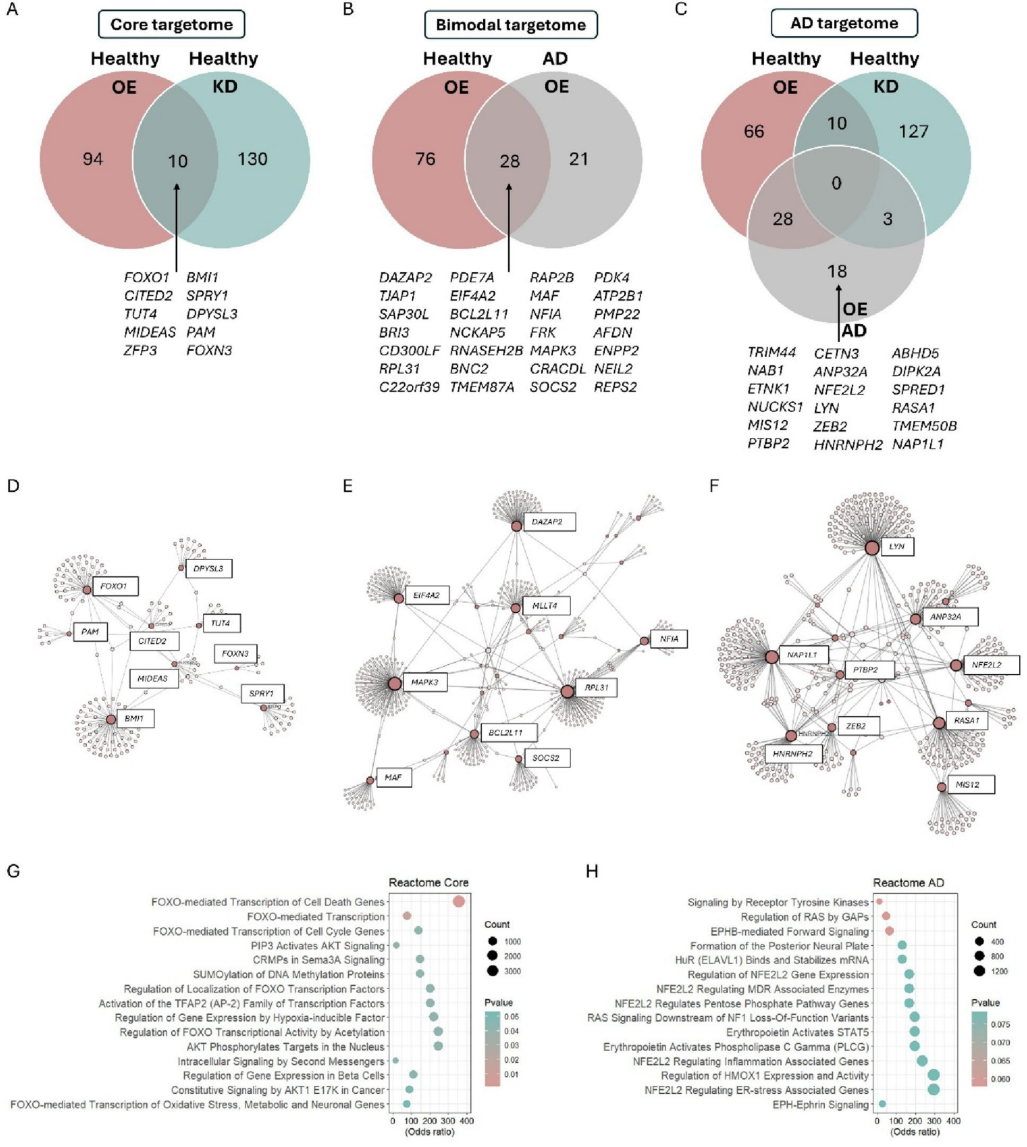
Identification of miR-132 targetome in microglia. **A** Overlap between putative miR-132 targets identified in healthy control iMGs after miR-132 overexpression and healthy control iMGs after knockdown of miR-132. **B** Overlap between putative miR-132 targets identified in healthy control and sAD iMGs after increasing miR-132 levels. **C** Overlap between putative miR-132 targets identified in all datasets analyzed. A full list of targets can be found in Supplementary Table 6. **D**–**F** IMEx Gene Network Analysis of the overlapping targets identified in Fig. 5A labelling all genes (**D**), Fig. 5B (**E**) and Fig. 5C (**F**) labelling genes with a betweenness centrality > 600.000 units (Supplementary Table 7. **G**, **H** Pathway enrichment analysis based on KEGG pathways for the miR-132 core targetome (**G**) and the AD-specific targets (**H**)

We next set out to identify putative miR-132 targets mediating the effects of miR-132 in healthy control and sAD iMGs. We first employed an in silico approach to extract predicted miR-132 targets, generating a list of 732 transcripts derived from a union of targets from three different miRNA target prediction algorithms, all carrying binding sites for miR-132 in their 3’-untranslated region (3’UTR) (TarBase, miRDB, miRDIP) (Supplementary Table 5). Next, for each of our transcriptomics datasets, all significant DEGs that were anticorrelated to the direction of the change of miR-132 levels were overlapped with the set of predicted targets, yielding 104 downregulated predicted targets after miR-132 supplementation in healthy control iMGs, 140 upregulated predicted targets after miR-132 knockdown in healthy control iMGs, and 49 downregulated predicted targets after increasing miR-132 levels in sAD iMGs (Fig. 5A–C, Supplementary Table 6).

To identify robust miR-132 targets in physiological conditions, we overlapped predicted targets from miR-132 overexpression and knockdown in healthy control iMGs, which yielded 10 targets identified in both datasets (Fig. 5A). To explore common networks in which these miR-132 core targets may be involved, we performed gene network analysis. Five of the miR-132 targets (*BMI1*, *FOXO1*, *SPRY1*, *DPYSL3*, *CITED2*) displaying the highest betweenness centrality, a proxy of network connectivity, have all shown to be involved in cell cycle, and more specifically, in neurogenesis [68–72] (Fig. 5D) (Supplementary Table 7). Additionally, multiple of these core miR-132 targets (e.g. *FOXO1*, *DPYSL3*, *SPRY1, BMI1, and CITED2* via *P300*) have previously been identified as miR-132 targets in other cell types, supporting the notion that they represent true miR-132 targets [2, 72–79]. GO term analysis further confirmed that the identified miR-132 core targetome is enriched for biological pathways that have been previously functionally linked to miR-132, like FOXO and Akt signaling [75, 76, 80, 81] (Fig. 5G).

Next, we identified 28 (downregulated) putative targets with a putatively bimodal behaviour, overlapping between healthy control iMGs and sAD iMGs upon miR-132 supplementation (Fig. 5B). Four of the overlapping putative targets with the highest betweenness centrality play a role in protein translation [82, 83] (*RPL3, EIF4A2*), MAPK signaling (*MAPK3*) and p53 signaling [84] (*DAZAP2*) (Fig. 5E) (Supplementary Table 7).

Lastly, we profiled AD-specific targets and found 10 putative miR-132 targets exclusively identified in sAD iMGs (Fig. 5C). Amongst these AD-specific targets were genes like *PTBP2*, a previously identified miR-132 target implicated in *Tau* mRNA splicing [85], *LYN*, a kinase shown to play a role in amyloid-β-triggered neurotoxicity and TAU hyperphosphorylation [86], and *NFE2L2*, *ZEB2*, *SPRED1* and *RASA1*, all previously shown to be regulated by miR-132 [87–89] (Fig. 5F). Pathway enrichment analysis confirmed that the identified AD-specific targets are functionally involved in AD-relevant pathways reported previously or in the current study, like Ras, NF-kB and TLR4 signaling [31, 80] (Fig. 5H).

Overall, our data indicate that miR-132 may have both ‘core’ targets, regulated independently of the cellular context, and context-dependent targets that differ between AD and healthy control iMGs.

### Neuronal miR-132 overexpression in AD mice exerts non-cell autonomous effects in a subpopulation of microglia

**Fig. 6:**
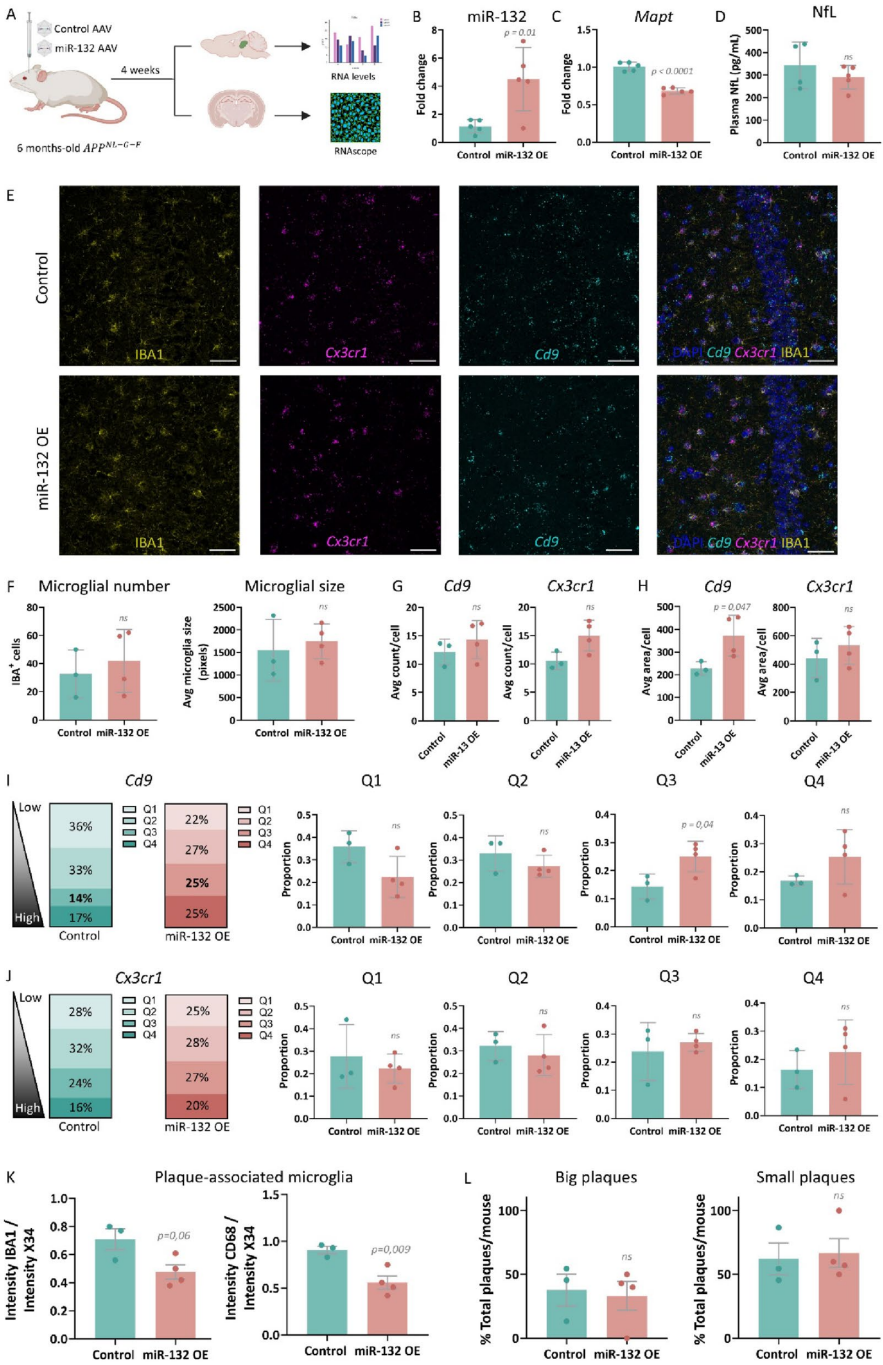
Targeted miR-132 overexpression in AD mouse brain. **A** Schematic representation of the experimental procedure employed for overexpression of miR-132 in hippocampal neurons in an AD mouse model. **B**, **C** Semi-quantitative real-time PCR of hippocampal miR-132 levels (**B**) and *Mapt* levels (**C)** upon miR-132 overexpression. n = 6 mice per group. Student’s t-test was used for statistical analysis. **D** Neurofilament light (NfL) levels as measured in blood plasma. n = 4–5 mice per group. Student’s t-test was applied for statistical analysis. **E** Representative images of the dentate gyrus in the brain of *App*^*NL−G−F*^ mice, immunolabeled for IBA1 and subjected to in situ hybridization for *Cd9* and *Cx3cr1* upon miR-132 or control AAV injection. Scale bars, 40 μm. **F** Total number of IBA^+^ cells and their size in the ROIs analyzed from the dentate gyrus. **G**, **H** Average transcript count (**G**) and average transcript area **(H)** per IBA^+^ cell of both *Cd9* and *Cx3cr1*. **I**, **J** Proportion of microglia present in different quartiles determined based on transcript count for *Cd9* (**I**) and *Cx3cr1* (**J)** comparing miR-132 overexpression and control group. In all graphs, student’s t-test was used for statistical analysis. **K** Quantification of IBA1 and CD68 intensity normalized to X34 intensity, around amyloid plaques. n = 3–4 mice per group. Student’s t-test was applied for statistical analysis. **L** Quantification of amyloid plaque (X34) abundance according to plaque size. n = 3–4 mice per group. Student’s t-test was applied for statistical analysis.

In vivo, microglia dynamically respond to cell-intrinsic and extrinsic signals derived from their tissue microenvironment. Having established the cell-intrinsic effects of miR-132 in iMGs in vitro, we next aimed to understand possible regulatory mechanisms in brain microglia triggered by miR-132. Gene targeting therapeutic strategies for neurological disorders using adeno-associated viruses (AAVs) with strong neuronal tropism are currently in clinical application [90, 91]. Such an approach could also be envisioned for miR-132, considering the numerous neuroprotective and neurotrophic regulatory effects it has in AD [20, 24, 30–32, 34, 92–95]. To address the potential of miR-132 as therapeutic target in vivo and assess putative non-cell autonomous effects of neuronal miR-132 in microglia, we employed a clinically relevant delivery system to increase miR-132 levels in hippocampal neurons. More specifically, we intrahippocampally injected a knock-in amyloidosis mouse model (*App*^*NL−G−F*^, MGI:5637817) with AAV.PHP.eB viruses under the control of the *Syn1* promoter, carrying either a miR-132 or control sequence cassette (Fig. 6A). We confirmed that miR-132 levels increased in the hippocampus (Fig. 6B), which led to the in vivo repression of one of the most often replicated neuronal miR-132 targets, *Mapt* [96] (Fig. 6C).

To monitor for any potential tissue toxicity induced by miR-132 overexpression, we measured the plasma levels of neurofilament light chain (NfL), a recognized biomarker for neurodegeneration [97]. There was no significant difference in NfL levels between miR-132 overexpression and control group, suggesting that the miR-132 overexpression did not induce neuronal damage (Fig. 6D). Additionally, based on histopathological assessment, no pathological microscopic findings related to miR-132 overexpression were observed, indicating that increasing miR-132 levels did not induce any major neurodegenerative or inflammatory changes (Supplementary Fig. 4).

Next, to investigate whether neuronal miR-132 overexpression may have non-cell autonomous effects on microglia, we quantified the expression levels of a marker for activated/disease-associated microglia [53, 55, 98, 99] (*Cd9*) and a marker for homeostatic microglia [53–57, 100–105] (*Cx3cr1*), by combining fluorescence in situ hybridization using RNAscope and immunohistochemistry for the general microglial marker IBA1 (Fig. 6E, Supplementary Fig. 5A). We validated the expression profile of *CD9* and *CX3CR1* in DAM and homeostatic microglia in a single-cell RNA sequencing dataset derived from human iPSC-derived microglia xenotransplanted in *App*^*NL−G−F*^ mice [53] (Supplementary Fig. 5B). First, we quantified the number and size of microglia present in the dentate gyrus and did not observe any significant differences between the miR-132 overexpression and control groups (Fig. 6F). While we did not find differences in *Cd9*^+^- and *Cx3cr1*^+^- counts per cell (Fig. 6G), we did observe a significantly higher *Cd9* area per cell, but no change for *Cx3cr1* (Fig. 6H). To probe microglial cell states, we categorized microglia into four quartiles based on the expression levels of *Cd9* or *Cx3cr1*, binning microglia with low expression levels in Q1 and high expression levels in Q4, for each of the two markers (Fig. 6I, J). Upon miR-132 overexpression, we found an increased proportion of microglia present in Q3 for *Cd9*, but no change in the proportions of microglia in any of quartiles for *Cx3cr1* (Fig. 6I, J).

We next assessed microglial recruitment to amyloid plaques by monitoring IBA1⁺-microglia and evaluated their lysosomal/phagocytic activation using CD68 expression as a readout [106] (Supplementary Fig. 5C). miR-132-injected hippocampi displayed a strong trend toward reduced IBA1⁺ intensity around plaques and a significant reduction in CD68⁺ intensity per plaque (Fig. 6K). However, CD68 expression per microglial cell remained unchanged around the plaques (Supplementary Fig. 5D). No alterations in the abundance of amyloid plaques were observed in miR-132-injected animals (Fig. 6L). These findings suggest that neuronally derived miR-132 may alter the activation state of specific microglial subsets in vivo and reduce plaque-associated microglial activation.

To assess the effect of AAV-mediated miR-132 supplementation at later stages of pathology, we measured the levels of TAU, Aβ_42_ (amyloid-β) and Aβ_40_ in the dentate gyrus of *App*^*NL−G−F*^ mice injected with the miR-132 or the control AAV at 11 months of age. miR-132 overexpression in hippocampal neurons significantly reduced the levels of TAU and Aβ_42_ compared to control-treated animals (Supplementary Fig. 5E), suggesting that miR-132 supplementation can have an impact on AD pathology.

Overall, we have established a safe delivery route for in vivo miR-132 overexpression in neurons, which can elicit non-cell autonomous effects in AD brain microglia. The relevance of these effects remains to be further elucidated.

## Discussion

In this study, we employed loss-of-function and gain-of-function approaches to investigate the cell-intrinsic regulatory mechanisms by which miR-132 regulates microglial gene expression and function in iMGs from both healthy controls and AD patients, and the cell-extrinsic mechanisms triggered in microglia by miR-132 in their tissue microenvironment in vivo. Although miR-132 was not essential for general immune response and phagocytotic capacity in healthy control iMGs, we report its regulatory impact over microglial gene expression in both healthy and AD conditions, and its dampening effect on the inflammatory response specifically in sAD iMGs. Additionally, we demonstrate that miR-132 overexpression in hippocampal neurons in a mouse model of AD shows a good safety profile and exerts non-cell autonomous effects on microglial states.

One of the primary functions of miRNAs is to maintain cellular homeostasis in the presence of cellular stressors, perturbagens or disease conditions [11, 107–109]. Yet, in physiological contexts, their individual impact may be less noticeable due to compensatory mechanisms and functional redundancy among several miRNAs and/or their targetomes [110], as it is exemplified by miRNA knockout models showing a phenotype only in combination with an additional stress-inducing factor [111, 112]. Indeed, we found that miR-132 loss-of-function in healthy control iMGs altered microglial gene expression without impacting their ability to elicit an inflammatory response or their phagocytic potential, while in iMGs derived from AD patients, a functional phenotype became apparent.

Another fundamental principle of miRNA biology is that miRNAs may not uniformly or simultaneously repress all their targets. Instead, most miRNAs prioritize their targets depending on the cellular context, the presence or absence of additional triggers, and the abundance of both other miRNAs and mRNA transcripts [12, 113, 114]. For instance, miRNA-mRNA networks in the central nervous system exhibit great specificity for distinct cell types, brain regions and developmental stages [115]. Our data indicate that while there are common ‘core’ miR-132 targets across conditions, possibly involved in ‘housekeeping’ cellular functions, like cell cycle [68–72], there are also context-dependent targets, specific to healthy control or sAD iMGs. A striking example is the regulation of complement component *C3*, a key mediator of neurodegeneration, that is secreted primarily by inflammatory microglia and increased in human AD brain [52, 116]. In healthy control iMGs, miR-132 loss-of-function decreased *C3* expression, while miR-132 supplementation increased it. In contrast, increasing miR-132 levels in sAD iMGs repressed *C3*. These opposing effects of miR-132 on *C3* could reflect a miR-132-target regulation paradigm that may have beneficial effects in AD where levels of *C3* and inflammatory genes were higher than in control conditions, but, notably, could instead be detrimental under healthy conditions.

Interestingly, we also identified miR-132 regulatory functions that could be putatively conserved across cell types. First, we observed that miR-132 depletion in healthy control iMGs altered gene expression of genes related to the regulation of neural stem cells and neuronal differentiation. This aligns with previous findings in mouse hippocampus, where miR-132 overexpression in neural stem cells modulated inflammatory signaling pathways, suggesting a prominent role for miR-132 in microglia-neuron crosstalk [31]. Additionally, miR-132 depletion in healthy control iMGs significantly downregulated *NOTCH1*, supporting earlier findings that demonstrated that Notch signaling is regulated by miR-132 in neural stem cells [44], and suggesting that this may be a cell type-independent function of miR-132. While Notch signaling might be instructive for microglial activation [117, 118], the implications of miR-132 regulation of *NOTCH1* in human microglia require further investigation. Lastly, miR-132 has been identified as a potent regulator of neural stem cell proliferation in the mouse dentate gyrus [31], and our data suggest a similar role in microglia, with miR-132 overexpression inducing several cell cycle-related pathways in healthy control iMGs. Notably, this effect on cell cycle was absent in sAD iMGs, further underscoring the context-dependent nature of miRNA regulation.

Multiple studies have confirmed that iPSC-derived microglia are transcriptionally similar to cultured primary human microglia, secrete cytokines in response to inflammatory stimuli and demonstrate phagocytic activity [45, 47, 51, 62]. Notably, iPSC-derived microglia with altered expression of the sAD risk genes *APOE4* and *TREM2* display pronounced phenotypic differences from control microglia, such as impaired phagocytosis, whereas mutations involved in familial AD have a more limited impact on microglial function [51, 60–63, 119–122]. Currently, evidence from iPSC-derived microglia obtained from sporadic AD patients is scarce [123]. Our findings demonstrate that sAD iMGs recapitulate transcriptional and functional profiles observed in human AD microglia. Notable shared features include reduced phagocytosis, previously reported in human iPSC-derived microglia with altered expression of AD risk genes [60–64], and increased expression of inflammatory genes (such as complement *C3)*, consistent with findings from human AD brain and APOE4/4 iPSC-derived microglia [52, 60, 61]. Interestingly, we did not observe significant differences when comparing miR-132 levels between AD and healthy control iMGs. Since miR-132 downregulation in AD has been only confirmed in neurons [2] and has yet to be extensively assessed in other cell types, our findings do not preclude that miR-132 levels may remain unchanged in microglia in the human AD brain. However, our sample size of two donors per group may have been insufficient to detect subtle differences, while more physiological conditions, such as co-cultures with other cell types [124], may be necessary to reveal such changes in future studies. Independently of whether miR-132 deficiency may actively contribute to microglia-related AD endophenotypes, miR-132 supplementation could be of therapeutic relevance; for example, even though evidence suggests that TREM2 levels do not change in human AD brain [125], therapeutic strategies to increase its function are being explored in clinical trials [126].

Anti-inflammatory properties of miR-132 have been demonstrated in multiple independent in vitro and in vivo studies, including its direct targeting of acetylcholinesterase (which leads to increased levels of acetylcholine and, thereby, inhibits the release of pro-inflammatory cytokines), and its regulation over key inflammatory pathways, like TLR4 and NF-kB, which we also observed in our study [41, 50, 76, 127–134]. Interestingly, miR-132-5p was recently reported to be among a small set of temporally responsive core miRNAs that drive the establishment and resolution of the inflammatory response upon exposure of healthy murine microglia to LPS [135]. However, a direct anti-inflammatory role in AD microglia has not been reported for miR-132 yet. Here, we show that miR-132 overexpression dampened the inflammatory response in sAD iMGs, potentially offering therapeutic benefits, as anti-inflammatory drugs are currently being evaluated in clinical trials for AD [136, 137].

Our findings point towards putative regulatory effects of miR-132 over microglial cell states with possible relevance for AD pathophysiology. miR-132 overexpression in AD iMGs significantly enriched signatures related to microglial activation and phagocytosis. This is a particularly interesting finding, given that we observed decreased phagocytic capacity in AD iMGs. Even though this induction of a more activated/phagocytic transcriptional microglial cell state did not result in a measurable effect on the phagocytic potential of the cells in vitro, it may still suggest that miR-132 activates an early transcriptional program that could eventually -under certain conditions- also have functional implications. Additionally, miR-132 attenuated pro-inflammatory cell state signatures that were significantly enriched in AD versus healthy microglia. These profiles correspond to DAM, inflammatory, interferon-responsive, antigen-presenting, cytokine-responsive, and senescence-associated microglia, all of which have been shown to be implicated in AD pathology. Apart from pathways related to inflammation, miR-132 supplementation in AD iPSC-derived iMGs also reversed signatures potentially associated with cellular fitness, like cell migration, survival and proliferation (that were predicted to be inhibited in AD and activated upon miR-132 overexpression); and apoptosis, necrosis (which were predicted to be enhanced in AD and repressed upon miR-132 overexpression).

We found *TLR4* to be significantly downregulated upon miR-132 supplementation in AD iMGs. Moreover, TLR4 signaling was enriched among the AD-specific miR-132 predicted targets. TLR4 levels are significantly higher in the brain of AD patients compared to controls [138]. In addition, TLR4 signaling has been shown to shift microglia toward an inflammatory state that is more disease-associated, ultimately compromising neuroprotection and cognitive function [139–143]. We also report that miR-132 represses NF-kB signaling in AD iMGs, another critical effector of Alzheimer's pathology and microglial function. Importantly, NF-kB also increases in AD brain [144], and NF-kB signaling modulates microglia by inducing a more inflammatory, disease-associated and cytotoxic state, while also contributing to TAU spreading [145, 146]. In addition to these critical effectors, several members of the AD-specific miR-132 predicted targetome that we identified, e.g. *TRIM44*, *NUCKS1*, *NFE2L2*, *LYN*, *ZEB2*, *SPRED1*, *ABHD5*, *RASA1*, and *NAP1L1* [143, 147–154], have known roles in inflammation and/or microglial cell states/function. Moreover, predicted miR-132 targets commonly downregulated in AD and healthy iMGs upon miR-132 supplementation have been previously identified as cytokine-response *(DAZAP2, PDK4, ENPP2*), DAM (*BRI3, RPL31, PDE7A, EIF4A2*) or other AD-related (*CD300LF, NCKAP5, RNASEH2B, BNC2, RAP2B, PMP22, REPS2*) markers in one or more datasets (Supplementary Tables 4, 6). Notably, the major signaling pathways that we found enriched among putative miR-132 targets (core & AD-specific targets), e.g. FOXO, Akt and Ephrin, have been shown to affect microglial phenotypes [155–157].

Furthermore, we confirm previous reports from studies in wild-type mouse brain microglia [42] by showing that miR-132 supplementation significantly represses AD risk genes in sAD iMGs. Analysis of the risk genes with the highest expression in our iMGs showed that the majority of the genes significantly downregulated by miR-132 (*MS4A6A, INPP5D, ACE, CR1*) are either increased in the human AD brain or their high expression levels were associated with increased risk of AD [158–161].

Microglia are a highly dynamic cell type that can undergo context-dependent functional adaptations [38]. Even though our data showed that neuronally targeted miR-132 overexpression in vivo induces an increase in the proportion of microglia highly expressing *Cd9* and limits amyloid plaque-associated microglial activation, additional analyses, such as single-cell RNA sequencing, would be needed to thoroughly profile microglial (subtype) responses on the molecular and functional level. Nevertheless, our data could provide a starting point for exploring a clinically relevant targeted delivery approach to increase miR-132 levels in neurons.

There are currently seven AAV-based gene therapy products that have received regulatory approval [91], and one AAV-based agent in a phase 2 clinical trial for AD (LX1001) [136]. AAV-mediated gene delivery is one of the most widely used strategies for in vivo overexpression of genes in the CNS, owing to its relatively low immunogenicity, stable expression, and broad tropism. However, several important limitations, like off-target effects due to ectopic or supra-physiological expression, immune responses and uneven or biased expression, constrain its effectiveness and interpretability -particularly in aged or inflamed CNS environments. The current study provides initial proof-of-concept that AAV-mediated neuronal delivery of miR-132 within the mammalian hippocampus can attenuate Aβ and TAU accumulation and modulate microglial activation, supporting its potential as a therapeutic strategy. The effects of miR-132 on phagocytic activation of AD microglia in vitro (enhanced phagocytosis-related transcriptional signatures in human iMGs) and in vivo (decreased phagocytosis-related activation of mouse microglia around amyloid plaques) could reflect differences between cell-autonomous and non-cell-autonomous signaling, species-specific programs or distinct microglial profiles engaging differentially towards soluble phagocytic cargos and already established amyloid plaques.

Our analysis revealed no evidence of toxicity, based on the biofluid marker NfL and histopathological analysis of the transduced tissue one month after local delivery of a miR-132-overexpressing AAV to the hippocampus. Previous studies have reported potentially harmful consequences of supra-physiological miR-132 levels on physiological functions [31, 92], which emphasize the need to establish a safe therapeutic window for miR-132. Although miR-132 supplementation appeared to have putatively beneficial effects in sAD iMGs, its impact in healthy control iMGs was more variable, showing bidirectional effects on inflammatory gene signatures.

**Fig. 7:**
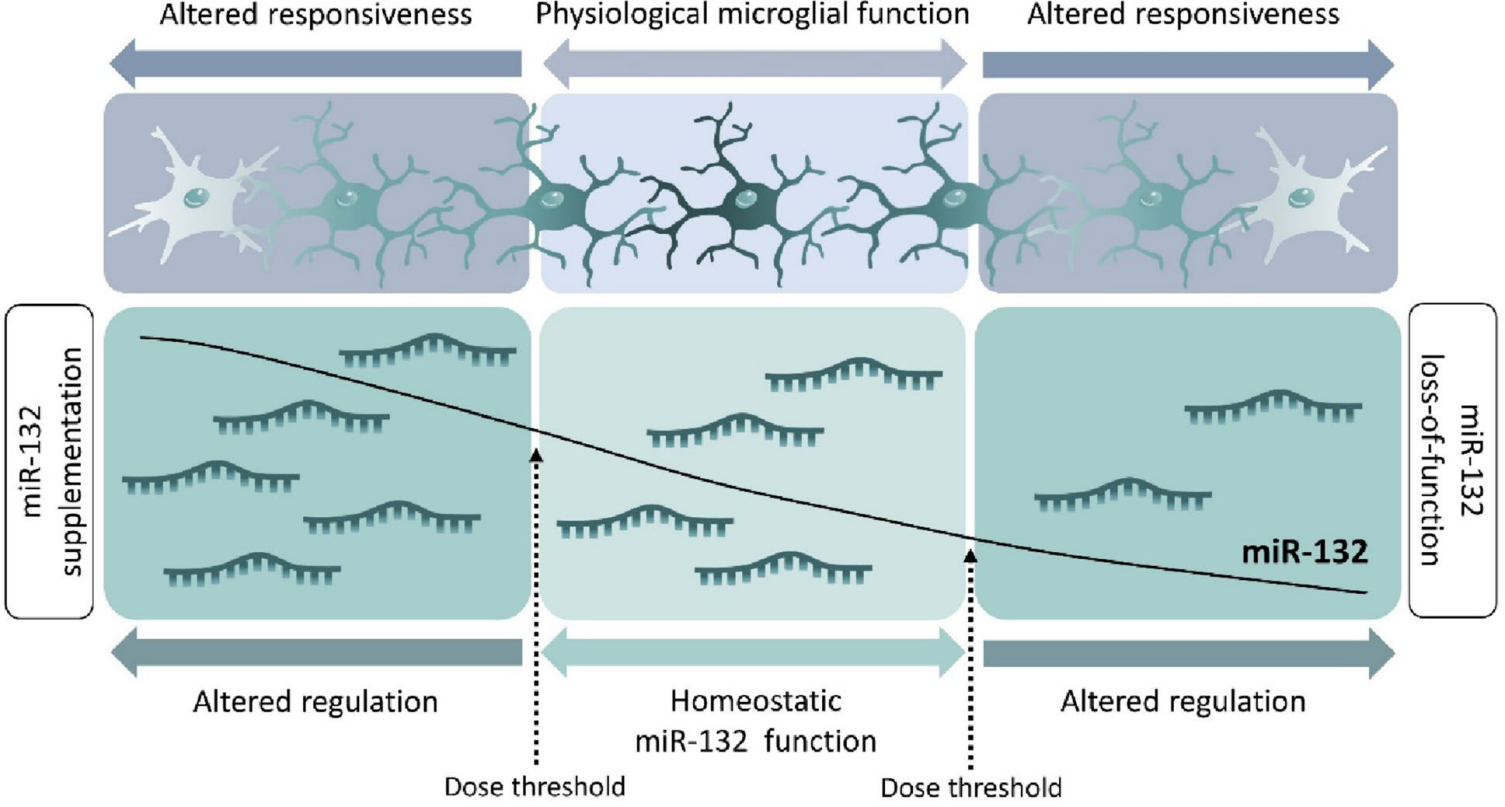
Schematic representation of a physiological window for optimal miR-132 expression and its potential effect on microglial responsiveness.

In summary, our data suggest that there is a certain range of miR-132 expression, within which it can perform its buffering functions to maintain microglial homeostasis under physiological conditions. This is an observation of particular clinical relevance: outside this ‘dose window’, there may be a biphasic response, with either non-sufficient or excessive expression of miR-132 and/or its targetome; aberrant gene expression regulation can subsequently impact the microglial inflammatory response (Fig. 7). Therefore, increasing miR-132 in AD may restore these altered molecular networks and biological pathways and thereby rescue some of the aspects of pathological microglial responsiveness.

The future of miRNA therapies in multifactorial neurodegenerative diseases, like AD, holds considerable potential but is limited by an incomplete understanding of their functions and specific roles in disease pathways. The pleiotropic nature of miRNAs also presents a challenge for therapeutic development, in particular given potential side effects. Furthermore, a lack of knowledge over miRNA sensitivity, specificity and selectivity towards their targets remains a major barrier to developing safe therapies, underscoring the need for improved strategies for targeted delivery. MiR-132 is no exception to this notion, and more systematic studies are required to map the breadth of its impact on AD pathophysiology. RNA-based therapies are gaining recognition as a new category of drugs with applications in numerous medical fields, including neurodegenerative disorders. The recent successful clinical trials using AAV-mediated gene delivery of a miRNA for the treatment of Huntington's disease may act as a catalyst for further investigation into the therapeutic potential of miR-132 in AD.

### Limitations

Here, we implemented genome-wide bulk RNA sequencing to globally profile miR-132-dependent alterations in transcriptional programs in healthy control and sAD iMGs. Increasing cellular and molecular resolution, with, for instance, single-cell RNA sequencing, could unveil more specific effects of miR-132 on microglial cell states in AD.

In addition, our findings cannot differentiate between direct and indirect targets of miR-132. While other approaches, like binding assays or miRNA pulldown methods, can probe direct interactions between miRNAs and mRNAs, they do not on their own offer evidence of functional relevance. Accounting for the complexity of miRNA biology, which can involve not only direct but also a series of indirect, similarly important, functional targets, we here aimed at mapping pertinent ‘endpoint’ transcriptional and functional readouts.

Another putative limitation could be the sample size of the iPSC experiments, where for some experiments two independent iPSC lines are used. Using multiple iPSC lines, rather than replicating the experiment within a single line, can increase statistical power [162]. However, it also introduces the risk of high inter-individual variability, as may have been occasionally the case in the present study.

Lastly, due to the current limitations in targeting microglia in vivo [163], we were not able to probe cell autonomous microglial effects of miR-132 in the AD mouse brain. In an alternative approach, we overexpressed miR-132 in neurons, where it was previously shown to be downregulated in the human AD brain, and profiled non-cell autonomous effects in microglia. Signaling through extracellular vesicles containing miR-132 from neurons to other cells has been shown to induce downstream effects [164–166], although elucidating the exact signaling mechanism in our experimental setup will require further research.

## Methods

### Induced pluripotent stem cell (iPSC) differentiation and culture

Induced pluripotent stem cells (iPSCs) were obtained from the Coriell Cell Repository (Camden, NJ) and previously characterized and genotyped by Meyer et al. [167]. The age at biopsy ranged from 64 to 75 years with similar gender representation (Supplementary Table 1).

iPSCs were differentiated into microglia as previously described [47]. In brief, iPSCs were thawed in a matrigel-coated 6-well plate (Corning Matrigel hESC-Qualified, Cat#354277) using mTeSR^TM^1 medium (Cat#85850, Stemcell), with the addition of 10 μM Y-27632 (ROCK inhibitor, Cat#72304, Stemcell) for the first 24 h. Once the cells reached confluence, they were transferred to an AggreWell™ 800 24-well plate (Cat#34815, Stemcell) to form Embryoid Bodies (EBs) at a density of 4 × 10^6^ cells per well. The EBs were fed daily by changing 75% of the medium with mTeSR1 containing 50 ng/mL Human BMP-4 Recombinant Protein (Cat#PHC9531, Thermo Fisher Scientific), 50 ng/mL Human VEGF-165 Recombinant Protein (Cat#100–20, Peprotech), and 20 ng/mL Human SCF (Cat#130-096-693, Miltenyi Biotec).

After four days, EBs were transferred to 6-well cell culture plates at a density of 10 to 20 EBs per well, containing X-VIVO15 (Cat#BE02-060F, Lonza) supplemented with 2 mM GlutaMAX™(Cat#35050038, Life Technologies), 50 μM β-mercaptoethanol (Cat#31350010, Thermo Fisher Scientific), 50 U/ml penicillin/streptomycin (Cat#P4333-100 mL, Sigma-Aldrich), 25 ng/mL Human IL-3 Recombinant Protein (Cat#PHC0035, Thermo Fisher Scientific), and 100 ng/mL Human M-CSF Recombinant Protein (Cat#PHC9501, Thermo Fisher Scientific). Half of the medium was replaced weekly.

After at least 6 weeks, macrophage precursors were collected using a 40 μm cell strainer, centrifuged at 400 g for 5 min, and resuspended in differentiation medium to generate microglia. The differentiation medium consisted of Advanced DMEM/F12 (Cat#12634010, Thermo Fisher Scientific) supplemented with 1 mM GlutaMAX, 50 μM β-mercaptoethanol, 50 U/ml penicillin/streptomycin, 100 ng/mL Human IL-34 Recombinant Protein (Cat#200–34, Peprotech), and 10 ng/mL Human GM-CSF Recombinant Protein (Cat#PHC2013, Thermo Fisher Scientific). Medium changes of 50% occurred three times per week.

iPSCs were differentiated into neurons as previously described [168]. Briefly, cells were cultured in Neural Maintenance Medium (480 ml Neurobasal medium (Fisher Scientific), 480 ml DMEM/F12 GlutaMAX (Fisher Scientific), 10 ml B27 supplement (Fisher Scientific), 5 ml GlutaMAX (Life Technologies), 5 ml Penicillin–Streptomycin (Sigma-Aldrich), 5 ml N2 supplement (Fisher Scientific), 1 ml 50 mM 2-mercaptoethanol (Fisher Scientific), 5 ml MEM Non-Essential Amino Acids (Fisher Scientific), 5 ml sodium pyruvate (Fisher Scientific), and 250 µl insulin (Sigma-Aldrich)), supplemented with 10 µM SB431542 (Sigma-Aldrich) and 1 µM LDN-193189 (Miltenyi Biotech) at 37 °C, 5% CO2 for ten days to induce differentiation into NPCs. Maturation of NPCs into neurons was induced by culturing the cells in Neural Maintenance Medium supplemented with 20 ng/mL brain-derived neurotrophic factor (BDNF; Stemcell Technologies), 20 ng/mL glial cell line-derived neurotrophic factor (GDNF; Stemcell Technologies), 200 µM cAMP (Stemcell Technologies) and 200 µM ascorbic acid (Sigma-Aldrich) on 0.01% poly-L-ornithine (PLO; Sigma-Aldrich) and laminin coated coverslips at 37 °C and 5% CO2.

### miR-132 oligonucleotide treatment of human iPSC-derived microglia

After 14 days of differentiation, iMGs were treated for miR-132 overexpression with a synthetic miR-132 mimic oligonucleotide (Batch ID 60374517, cholesterol-conjugated, Janssen Pharmaceutica) or a corresponding control oligonucleotide, consisting of C. elegans miR-67 with the same modifications as the mimic, at 1500 nM, and after 1 week of treatment they were either collected in 700 µl QIAzol (QIAGEN) for RNA extraction or used for functional assays and then collected as described below. For miR-132 knockdown, differentiated iMGs were treated with a synthetic miR-132 antisense oligonucleotide (cholesterol-conjugated, Cat#339408, QIAGEN) or corresponding control oligonucleotide at 1500 nM, and after 24 h they were either collected in 700 µl QIAzol Lysis Reagent (Cat#79306, QIAGEN) for RNA extraction or used for functional assays.

### RNA extraction, reverse transcription and semi-quantitative real-time PCR

RNA isolation from iMGs was performed using the miRNeasy Micro Kit (Cat#217084, Qiagen) following manufacturer’s instructions. Briefly, cells were homogenized in QIAzol, followed by a 5-min incubation in chloroform at RT. After 15 min of centrifuging at 12,000 g at 4 °C, the upper aqueous phase was collected and supplemented with 1.5 volumes of 100% ethanol and loaded on RNeasy MinElute spin columns. Following washing steps, RNA was eluted in 14 µl of RNase-free water and measured using the NanoDrop ND-1000 Spectrophotometer (NanoDrop Technologies, Inc.).

RNA isolation from the mouse hippocampus was performed using the miRVana Paris Kit (Cat#AM1556, Life Technologies) according to the manufacturer’s instructions. Briefly, samples were homogenized using 350 μl of cell disruption buffer containing protease and phosphatase inhibitors. After denaturation, acid-phenol:chloroform was added and the samples were incubated and spun down, resulting in the formation of 2 phases. The upper aqueous phase was collected and supplemented with 1.25 volumes of 100% ethanol and loaded on miRVana spin columns. Following consecutive washing steps, RNA was eluted from the column in 30 µl of elution solution and concentration and purity were measured using the NanoDrop ND-1000 Spectrophotometer (NanoDrop Technologies, Inc.). Three females and two males were used for the control group, and two females and three males for miR-132 overexpression.

Reverse transcription of miRNAs was performed with 100 ng RNA using the miRCURY LNA RT Kit (Cat#339340, QIAGEN). For the reverse transcription of mRNAs, 200 ng RNA was used to synthesize cDNA using the Superscript II reverse transcriptase (Cat#18064071, Thermo Fisher Scientific).

For miRNAs, real-time semi-quantitative PCR was performed using the miRCURY LNA SYBR Green PCR Kit (Cat#339345, QIAGEN) together with miRCURY LNA primers (Cat#339306, QIAGEN). For normalization, the mean expression of small-RNA housekeeping genes *Rnu1a1* (Cat#YP00203909, QIAGEN) and *Rnu5g* (Cat#YP00203908, QIAGEN) were used for mouse miRNA and *SNORD48* (Cat#YP00203903, QIAGEN) and *RNU5G* (Cat#YP00203908, QIAGEN) for human miRNA. For mRNA transcripts, real-time semi-quantitative PCR was performed using the PowerUp™ SYBR™ Green Master Mix (Cat#A25776, Bioline Applied Biosystems™) and *RPLP0* and *UBC* were used as housekeeping genes for human mRNAs (Supplementary Fig. 2E). *Gapdh* and *Actin* were used as housekeeping genes for mouse mRNAs. Primer sequences can be found in Supplementary Table 8. Ct values were determined using the second derivative method and subsequently fold changes were calculated using the ΔΔCt method.

### Bulk RNA-sequencing library preparation, sequencing, and data pre-processing

iMGs were collected and RNA was extracted as described above. RNA purity was measured with the 4200 TapeStation (Agilent Technologies) and 200 ng RNA was used as input for library construction. All samples were sequenced in one batch. Libraries were constructed using the DNBSEQ platform at BGI (BGI Genomics, China) following the manufacturer's instructions, generating 24 million clean,

150 bp paired-end reads per sample. Raw sequencing reads were assessed for quality using FastQC (version 0.11.9), and an overview of quality metrics across all samples was generated using MultiQC (version 1.15.dev0). High-quality reads were aligned to the reference genome GRCh38.p13 using the STAR aligner (version STAR-2.7.10b) with default settings. Alignment metrics, including mapping rate and uniquely aligned reads, were assessed using MultiQC. Aligned reads were counted using *featureCounts* from the Subread package (version 2.0.2). Counts were assigned to annotated features based on the GENCODE v42, and only reads overlapping exonic regions were included in downstream analyses.

### Bulk RNA sequencing differential expression analysis

Gene-level count data were analyzed for differential expression using DESeq2 (version 1.42.0).

For sAD vs healthy control comparisons, technical replicates were collapsed into a single sample with *collapseReplicates* function from DESeq2 resulting in 2 biological replicates for AD lines and 3 biological replicates for healthy control lines. The count data were filtered to retain only genes with a total read count of at least 10 across all samples. For normalization and differential expression analysis, the standard DESeq2 pipeline was applied. Count data were normalized using the median-of-ratios method, and differential expression between AD and healthy control samples was assessed using the Wald test. Results were ranked based on adjusted p-values, and significance was determined using an adjusted p-value cut-off of 0.05. Genes with an absolute log2 fold change greater than 0.25 and an adjusted p-value lower than 0.05 were considered significantly differentially expressed. For comparisons, where insufficient number of biological replicates was available for treatment and control groups, technical replicates were accommodated using the *duplicateCorrelation* function from the limma (version 3.58.1) package. Count data was first normalized using the TMM (trimmed mean of M-values) method, and the voom transformation was applied to estimate the mean–variance relationship in the data. To account for the correlation between technical replicates, *duplicateCorrelation* was run to estimate the intra-group correlation, and this value was incorporated into the linear modeling step. A second voom transformation was performed with the estimated correlation to refine the weights. Linear modeling was conducted using *lmFit* with the correlation structure applied, followed by empirical Bayes moderation of the standard errors via *eBayes* function. Genes with an absolute log2 fold change greater than 0.25 and an adjusted p-value lower than 0.05 (adjusted by the Benjamini–Hochberg method) were considered significantly differentially expressed.

### Pathway and gene set enrichment analysis

To identify biological pathways on which miR-132 regulation converges, we used gene ontology (GO) term enrichment analysis using the Database for Annotation, Visualization, and Integrated Discovery [169, 170] (DAVID). For all comparisons, the input was all significant DEGs that were either upregulated or downregulated. As background list, all genes were used that were identified by bulk RNA sequencing and had a read of at least 10 across all samples. GO Biological Process Direct pathways were ranked based on their significance, and pathways with a p-value below 0,05 were selected for analysis. The top 15 significant pathways were used as representation in the figures. Pathway enrichment among the miR-132 core and AD-specific targetome was additionally evaluated using EnrichR (maayanlab.cloud/Enrichr/) with the Reactome 2024 pathway database. Predicted miR-132 core or AD-specific targets (Fig. 5A, C and Supplementary Table 6) were submitted through the EnrichR web interface, and enrichment scores and adjusted *p*-values were obtained using the platform’s default statistical methods. For computing and visualizing ‘functional’ enrichment of microglial cell state signatures in AD versus healthy and miR-132 overexpression versus control in AD iMGs, the stats (version 4.3.3, phyper) and pheatmap (version 1.0.13) packages in R were used respectively.

Gene Set Enrichment Analysis (GSEA) was performed with the lists in Supplementary Table 4, using the weighted enrichment statistic and ‘Diff_of_Classes’ as ranking metric.

### Ingenuity pathway analysis (IPA)

#### Dataset preparation and upload

Differential expression datasets from (1) AD miR-132 overexpression (OE) versus AD control and (2) AD versus healthy control were generated and imported into Ingenuity Pathway Analysis (IPA; Qiagen) as separate datasets. For each dataset, gene identifiers, fold-change values, and corresponding p-values were uploaded using IPA’s Upload Dataset function.

#### Core analysis

A Core Analysis was performed independently for each dataset. Analyses included Upstream Regulator Analysis and Diseases & Functions. Activation z-scores were calculated by IPA using the Ingenuity Knowledge Base as reference.

#### Upstream regulator filtering and miR-132 predicted targets overlay

The list of predicted upstream regulators from each core analysis was exported. To identify miR-132-related regulatory effects, this list was cross-referenced with validated and predicted miR-132 targets (Supplementary Table 5). Regulators predicted by IPA to be inhibited (negative activation z-score ≤ –2) in the AD miR-132 OE versus AD control dataset were retained for downstream interpretation.

#### Comparison analysis

A Comparison Analysis was conducted between the two datasets (AD miR-132 OE versus AD control and AD versus healthy) to evaluate how miR-132 overexpression influenced disease-related transcriptional signatures. IPA’s comparison tool was used to contrast activation z-scores across datasets.

#### Disease and functional reversion analysis

To examine whether miR-132 overexpression counteracted AD-associated dysregulation, Diseases & Functions terms were evaluated for changes in predicted activation state between datasets. Functions with opposite activation patterns (e.g., activated in AD versus healthy but inhibited in AD miR-132 OE versus AD control) were annotated as exhibiting reversion toward a healthy state.

### Microglial functional assays

To assess the effect of miR-132 on microglial function, iMGs were cultured and treated with oligonucleotides as described above and used for functional assays on the day of collection. To induce inflammation, iMGs were treated with 10 ng/ml LPS (Cat#L4516, Sigma) for 6 h and then collected in QIAzol. To assess phagocytosis, iMGs were seeded onto coverslips and treated with fluorescent latex beads (Cat#L1030, Sigma) at a concentration of 0,01% beads in medium for 90 min. Subsequently cells were fixated in 4% PFA (Cat# J19943.K2, Thermo Fisher) for immunolabeling.

### Enzyme-Linked Immunosorbent Assay (ELISA)

Conditioned medium was collected after 6 h of LPS treatment. After a 10-min centrifugation at 1500 rpm at 4°C, supernatants were collected and stored at -20°C until use. Subsequently, standards and samples were loaded on the same plate and probed using the V-Plex proinflammatory panel 1 human kit (Cat# K15049D, Meso Scale Discovery, MSD). Plates were recorded on an MSD microplate reader (MESO SECTOR S 600).

For Aβ and TAU detection in dentate gyrus homogenates from 12-month old *App*^*NL−G−F*^ animals (injected at 11 months of age as described under ‘Intracranial injections’), the monoclonal antibodies JRFcAβ40/28, JRFcAβ42/26 (which recognize the C terminus of Aβ species terminating at amino acid 40 or 42, respectively) and JRD/PT/51 (Janssen R&D), were used as capture antibodies. JRFAβN/25-sulfo and JRD/PT/82-sulfo (Janssen R&D) were used as the detection antibodies, respectively. Plates were recorded on an MSD microplate reader as before. The control group included three females and five males, and the miR-132 overexpression group included three females and three males.

### Immunocytochemistry and image analysis in cell lines

iPSCs and iMGs were PFA-fixed on coverslips, then permeabilized in 1% (v/v) Triton X-100 in 1X PBS, which was followed by 2 h of room temperature (RT) incubation with blocking buffer, consisting of 1% (v/v) Triton X-100 and 10% (v/v) normal goat serum in 1X PBS. Primary antibody incubation was performed in 0.3% (v/v) Triton X-100, 3% (v/v) normal goat serum in 1X PBS at 4 °C overnight in a humidified chamber. This was followed by incubation in secondary antibody for 2 h at RT. Finally, cells were stained with DAPI and mounted in Mowiol. All antibodies used are listed in Supplementary Table 9. For quantification of phagocytosis, images were acquired at 20X magnification, with four fields imaged per coverslip, and three coverslips analyzed per condition. Images were processed using FIJI, applying a consistent binarization threshold across all images to ensure uniformity. After binarization, we utilized CellProfiler for detailed analysis, segmenting the cells based on IBA1 and DAPI staining to clearly delineate cell boundaries. Within these segmented cells, we quantified the number of fluorescent beads.

### Target prediction

In silico miR-132 target prediction was performed by using the miRNA target prediction tools TarBase [96], miRDB [171, 172], and miRDIP [173, 174]. For all prediction tools, targets were filtered based on Homo Sapiens genome. TarBase was additionally filtered based on brain tissue and HITS-CLIP data. miRDB targets were used with a Target Score of 70 or higher and miRDIP targets were used with an Integrated Score of 0.6 or higher. The full list of miR-132 targets used in this study can be found in Supplementary Table 5.

### Network analysis

Gene network analysis was performed using the Network Analysist platform [175]. Protein–protein interactions were identified using the IMEx Interactome as database [176], building a first-order network. Full results of the gene network analysis can be found in Supplementary Table 7.

### Animals

Mice were bred following standard laboratory procedures and kept under a 12-h light–dark cycle. Food and water were available ad libitum. The AD mouse models utilized in this study were *App*^*NL−G−F*^ animals (MGI:5637817, Takaomi Saido, RIKEN Brain Science Institute, Japan). All animal experiments were approved by the ethical committees of the Royal Dutch Academy of Arts and Sciences (AVD80100202010904) and complied with European guidelines for the care and use of laboratory animals (Council Directive 86/6009/EEC).

### Intracranial injections

Intracranial injections were performed with the following stereotactic coordinates: AP -2.00 mm, ML + 1.50 mm or -1.50 mm depending on hemisphere, DV -1.90 mm (from the skull). For miR-132 overexpression, male and female 6-month-old *App*^*NL−G−F*^ mice (or 11-month old for ELISA measurements) were injected with syn:AAV.PHP-eB:GFP harbouring a miR-132 hairpin sequence (VectorBuilder, Germany). As negative control, hairpin constructs corresponding to cel-miR-67 were used. For both viruses, 1 µl (8 × 10^13^ TU/ml for 6-month old mice; 4 × 10^12^ TU/ml for 11-month old animals) was injected at 0.2 µl/min in each hemisphere and mice were sacrificed 4 weeks post injection (or 3 weeks for the 11-month old animals). Older animals were additionally perfused with saline before sacrifice. Tissue samples for RNA isolation were snap-frozen and stored at -80 °C prior to processing. Mice used for fluorescence in situ hybridization were perfused with cold 4% PFA and tissue was directly fixed in cold 4% PFA overnight, then cryopreserved by immersion in 15% and then 30% sucrose in 1X PBS until sunken, and frozen and stored at – 80 °C. Brains used for histology were collected and preserved in 10% neutral buffered formalin and stored at RT.

### Neurofilament light quantification

Blood was obtained by heart puncture and collected in a K2 EDTA prepared Microvette (Cat#16.444.100, Sarstedt), then centrifuged at 2000 × g for 10 min to obtain plasma. Plasma samples were then stored at − 80 °C until use. Neurofilament light levels were measured with an electrochemiluminescence immunoassay using the R-PLEX human Filament L Kit (Cat#K1517XR-2, Mesoscale) according to manufacturer’s instructions.

### Histology assessment

Brains were embedded in paraffin, sectioned, and mounted on glass slides. All slides were stained with hematoxylin and eosin. Samples were evaluated histopathologically by light microscopy by a board-certified veterinary pathologist.

### RNAscope in situ hybridization and quantification

RNAscope in situ hybridization (ISH) was performed according to manufacturer instructions. Sections were cut at a cryostat at 16 μm thickness and dried on slides for 1h at -20°C before being stored at -80°C until use. Briefly, tissue was washed in cold 1 × PBS (5 min) and baked for 30 min at 60°C. Sections were fixed in cold 4% PFA for 15 min, then dehydrated in a gradient of ethanol (50%, 70%, 100%) for 5 min each and air-dried for 10 min. Once dried, hydrogen peroxide (Cat#322381, BioTechne) was applied for 10 min and sections were immediately washed with nuclease-free water (NFW) for 2 × 5 min. For target retrieval, sections were kept in boiling anti-retrieval solution (Cat#322000, BioTechne) for 15 min, after which sections were washed 2 × 5 min in NFW, then rinsed in 100% ethanol and airdried. Sections were then incubated with Protease III (Cat#322381, BioTechne) for 10 min at 40 °C, and rinsed 2 × 5 min with NFW. RNAscope probes for *Cd9* (Cat#430631-C3, BioTechne) and *Cx3cr1* (Cat#314221-C2, BioTechne) were added and hybridized for 2 h at 40 °C. After hybridization, sections were washed in Wash Buffer (Cat#310091, BioTechne) and stored in 5 × SSC-buffer (Cat#S6639, Merck) overnight at RT. For amplification, sections were incubated consecutively with AMP1, AMP2 and AMP3 (Cat#322780, BioTechne) for 30 min at 40°C with washes between AMP reagents. The *Cx3cr1* probe signal was developed with the horseradish peroxidase (HRP) for C-2, and afterwards with TSA Vivid 650 (Cat#323273, BiotTechne), for 15 min and 30 min respectively at 40 °C. After incubation with HRP-blocker (Cat#322780, BioTechne) for 15 min at 40 °C, probe development was repeated for *Cd9* with HRP-C3 and TSA Vivid 570 (#323272, BioTechne). We used one female and two male animals in the control group, and three females and one male in the miR-132 overexpression group.

RNAscope ISH was followed by fluorescent labeling of IBA1 protein using immunolabeling, as described above, but without the blocking step. Sections were counterstained with DAPI (Cat#322750, BioTechne) for 1 min and mounted with ProLong Gold Antifade Mountant (Cat#P36930, Thermo Fisher Scientific). Slides were dried in the dark at RT for at least 4 h and stored at 2–8 °C until imaged with a Leica TCS SP8 confocal system.

Images acquired were analyzed using the ImageJ [92], CellPose [177] and CellProfiler [178] software. Using the ImageJ software, ROIs in the dentate gyrus were extracted, of the same size and spatial concordance in both experimental groups. More specifically, the anatomical features of the granule cell layer of the dentate gyrus depicted by the DAPI channel were used to draw a line from the bifurcation point of the upper and deeper arms of the dentate gyrus until the lateral-most “tip” of the upper arm. The information from this line was used to transform and rotate the image, and calculate the x-coordinates for the placement of the ROIs, relative to each imaged hippocampus. The y-coordinates were calculated based on a line extending from the upper-most part of the upper arm of the dentate gyrus until the lower-most “tip” of the deeper arm. Thereafter, the x- and y- coordinates were accordingly calculated for each hippocampus imaged, and three ROIs were placed along these axes. Each ROI consisted of 800 × 800 pixels and entailed a portion of the hilus and the molecular cell layer region, with the granule cell layer centered within the ROI. IBA1-channel ROIs underwent global thresholding to create a binary image. DAPI-channel ROIs were segmented using the CellPose software and binarized using the ImageJ software. Lastly, using the CellProfiler software a pipeline was generated to identify IBA^+^ cells overlaying with DAPI and extract the transcript information of the *Cd9*- and *Cx3cr1*- channels within these cells (Supplementary Fig. 5).

### Immunofluorescence and image analysis in mouse brain

For mouse brain immunofluorescence, 16 µm-thick cryostat-prepared coronal tissue sections were initially permeabilized in 1% (v/v) Triton X-100, followed by 2 h blocking in 1% (v/v) Triton X-100, 10% (v/v) normal goat serum in PBS at RT. Primary antibody incubation was performed in 0.3% (v/v) Triton X-100, 3% (v/v) normal goat serum overnight at 4 °C in a humidified chamber, and incubation in secondary antibody for 2 h at RT. To label Aβ plaques, sections were incubated for 20 min at RT in 20 μM X34 (Sigma-Aldrich) dissolved in 60% PBS/40% ethanol (pH 10). Finally, sections were mounted with Mowiol. All the antibodies used for immunolabeling of brain tissue are listed in Supplementary Table 9.

Fluorescence was quantified from fluorescence microscopy images using ImageJ software. The area surrounding each plaque was defined with the circle selection tool, ensuring inclusion of the X34⁺ plaques and the associated IBA1⁺ cells. For each plaque, the area, integrated density, and mean gray value were measured for the X34, IBA1, and CD68 channels using the Analyze → Measure function in ImageJ. Background mean gray values were obtained in the same manner from nearby regions lacking signal.

Corrected total cell fluorescence (CTCF) was calculated using the standard formula:

$$ {\mathrm{CTCF}} = {\mathrm{Integrated}}\;{\mathrm{Density}} - \left( {{\mathrm{Area}}\;{\mathrm{of}}\;{\mathrm{selection}} \times {\mathrm{Mean}}\;{\mathrm{background}}\;{\mathrm{fluorescence}}} \right) $$

Plaque size was quantified by calculating the mean, maximum, and minimum areas of all plaques. Plaques with areas between the mean and maximum were classified as ‘big plaques,’ whereas those with areas between the mean and minimum were classified as ‘small plaques.

### Statistics

Statistical analysis was conducted as described in the figure legends using GraphPad Prism, with significance defined by an adjusted p-value threshold of 0.05. Normality was evaluated using the Kolmogorov–Smirnov test; non-normally distributed data were analyzed using the Mann–Whitney U test. Data in the graphs are presented as mean ± standard deviation.

## Supplementary Information

Below is the link to the electronic supplementary material.

Supplementary Material 1

Supplementary Material 2

Supplementary Material 3

Supplementary Material 4

Supplementary Material 5

Supplementary Material 6

Supplementary Material 7

## Data Availability

All data needed to evaluate the conclusions in the paper are present in the paper. The RNA sequencing data of this study will be available at GEO upon publication.

## Abbreviations

AD: Alzheimer’s disease
miRNA: MicroRNA
miR-132: MicroRNA-132
iPSC: Induced pluripotent stem cell
iMG: iPSC-derived microglia
OE: Overexpression
KD: Knockdown
GO: Gene ontology
LPS: Lipopolysaccharide
GSEA: Gene set enrichment analysis
3’-UTR: Three-prime untranslated region
AAV: Adeno-associated virus
NfL: Neurofilament light chain
