# Supplementary material for: microRNA-132 attenuates inflammation in induced pluripotent stem cell-derived microglia from Alzheimer’s disease patients
Source: Acta Neuropathol Commun. 2026 Mar 7;14:99. doi: 10.1186/s40478-026-02228-8 (PMC13104464; doi:10.1186/s40478-026-02228-8)
Supplement: Supplementary file 1 — Supplementary Material 1 [file 40478_2026_2228_MOESM1_ESM.pdf]

Supplementary Figures and Tables

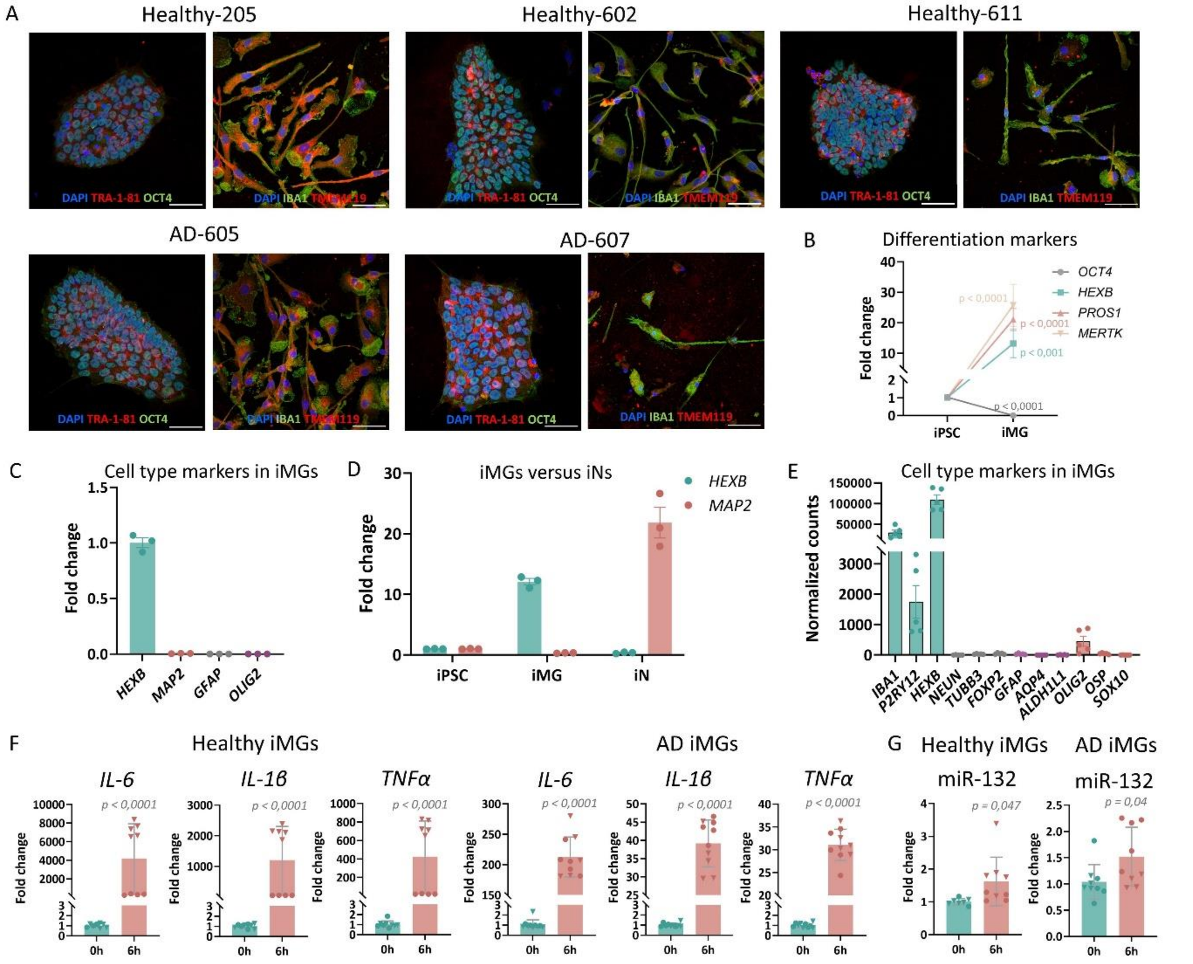

Supplementary Figure 1. Characterization iPSC-derived microglia-like cells

(A) Representative images of immunolabeling of TRA-1-81^{+} and OCT4^{+} iPSC colonies and IBA1^{+} and TMEM119^{+} iMGs. Scale bars, 50 µm. (B) Semi-quantitative real-time PCR of *OCT4*, *HEXB*, *PROS1* and *MERTK* in iPSCs and iPSC-derived microglia. n = 3 healthy iPSC lines and 2 AD iPSC lines. (C) Semi-quantitative real-time PCR of *HEXB*, *MAP2*, *GFAP* and *OLIG2* in healthy iPSC-derived microglia. n = 3 technical replicates. (D) Semi-quantitative real-time PCR of *HEXB* and *MAP2* in healthy iPSCs, iPSC-derived microglia and iPSC-derived neurons (iN). n = 3 technical replicates. (E) Normalized RNA-sequencing read counts of a panel of cell type markers in iPSC-derived microglia. n = 3 healthy iPSC lines, 2 AD iPSC lines. (F) Semi-quantitative real-time PCR of *IL-6*, *IL-1β* and *TNF-α* levels after LPS stimulation in mature iMGs. n = 5 technical replicates per iPSC line; 2 iPSC lines in total. Mann-Whitney U test was applied for statistical analysis of Healthy iMGs. Student's t-test was applied for statistical analysis of AD iMGs. (G) Semi-quantitative real-time PCR of miR-132 in mature iMGs. n = 4-5 technical replicates per iPSC lines; 2 iPSC lines in total. Student's t-test was applied for statistical analysis. Data point symbols indicate the origin of the iPSC lines.

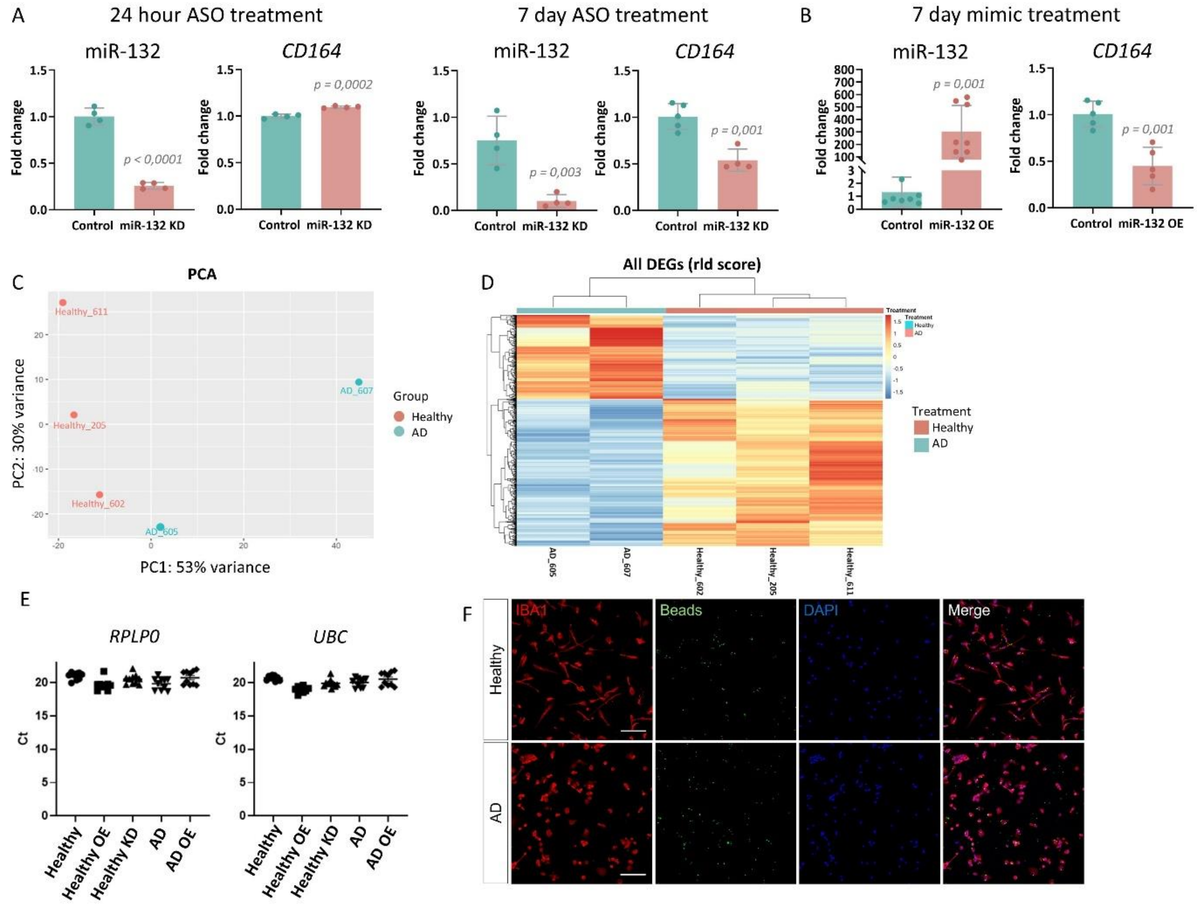

Supplementary Figure 2. Oligonucleotide treatment optimization and comparative baseline iMG analysis

(A) Semi-quantitative real-time PCR of miR-132 and *CD164* in iPSC-derived microglia after 24-hour or 7-day treatment with a miR-132 antisense oligonucleotide. n = 4 technical replicates; 1 iPSC line. Student's t-test was applied for statistical analysis. (B) Semi-quantitative real-time PCR of miR-132 and *CD164* in iPSC-derived microglia after 7-day treatment with a miR-132 mimic oligonucleotide. n = 3-4 technical replicates; 2 independent experiments; 1 iPSC line. Student's t-test was applied for statistical analysis. (C) Principal component analysis (PCA) of iMGs based on gene expression (bulk RNA-seq). Axes values indicate the percentage variance. (D) Heatmap of all differentially expressed genes (DEGs), visualized using regularized log transformed values. (E) Semi-quantitative real-time PCR of housekeeping genes *RPLP0* and *UBC*. One-way ANOVA with Bartlett's post hoc correction was applied for statistical analysis. Ct, cycle threshold. (F) Representative confocal images of immunolabeled iMGs for phagocytosis assay quantification. Scale bars, 100 µm.

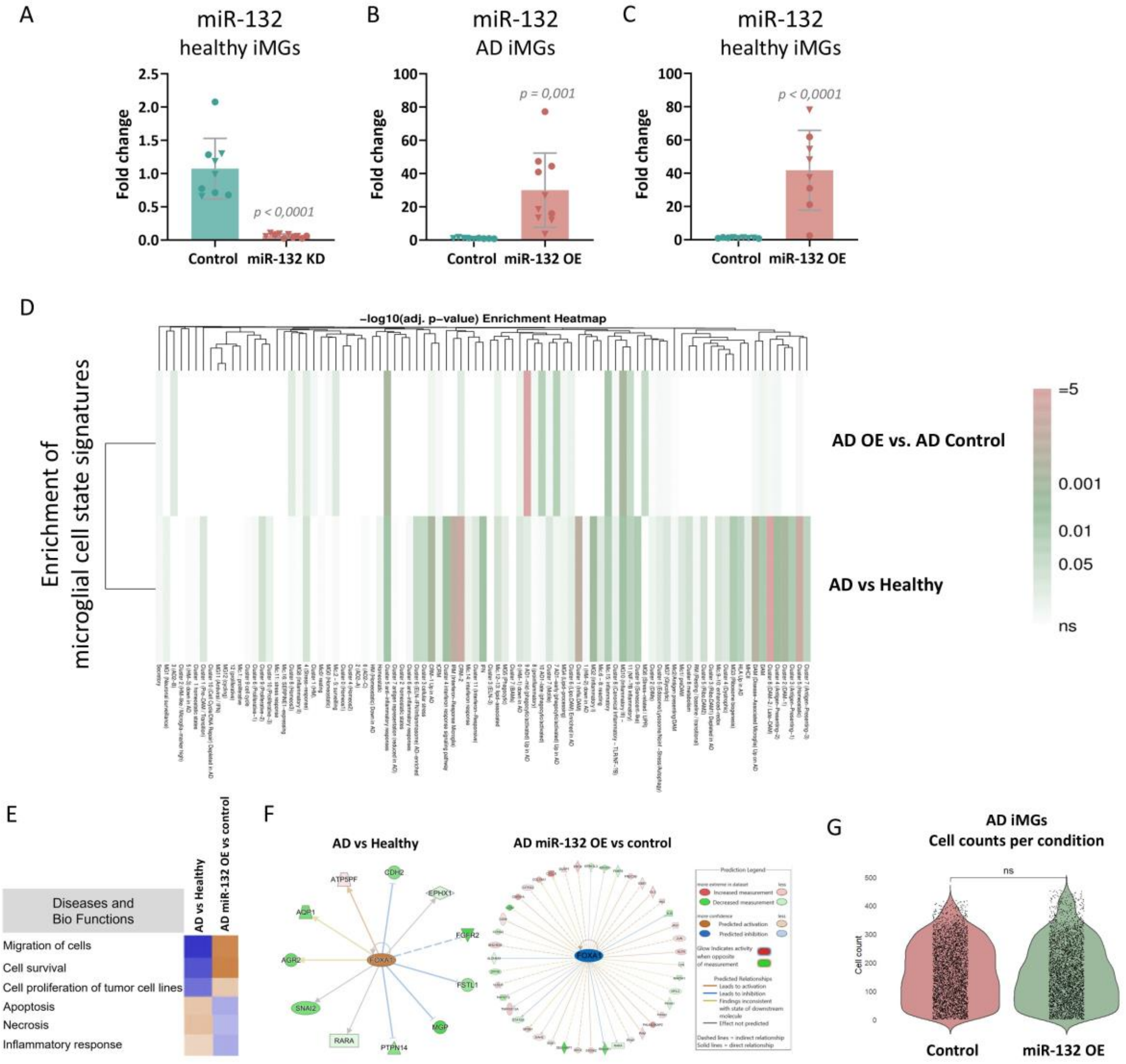

Supplementary Figure 3. miR-132 knockdown and supplementation in iMGs

(A-C) Semi-quantitative real-time PCR of miR-132 in iPSC-derived microglia before LPS assay, after miR-132 knockdown in healthy iMGs (A), miR-132 overexpression in AD iMGs (B), and miR-132 overexpression in healthy iMGs (C). n = 4-5 technical replicates; 2 iPSC lines in total. Student's t-test was applied for statistical analysis. Data point symbols indicate the origin of the iPSC lines. (D) Enrichment analysis of microglial cell state signatures comparing AD iMGs OE versus control and AD versus healthy iMGs. (E) Ingenuity Pathway Analysis (IPA) of DEGs in AD iMGs compared to healthy control iMGs, and miR-123 OE versus control in AD iMGs. (F) IPA regulator analysis for FOXA1. (G) Quantification of cell density in AD iMGs in the control group and miR-132 OE group, as assessed by the number of IBA1-positive cells. n = 3846 cells in control and 3614 cells in miR-132 OE, 12 images per condition. Student's t-test was applied for statistical analysis.

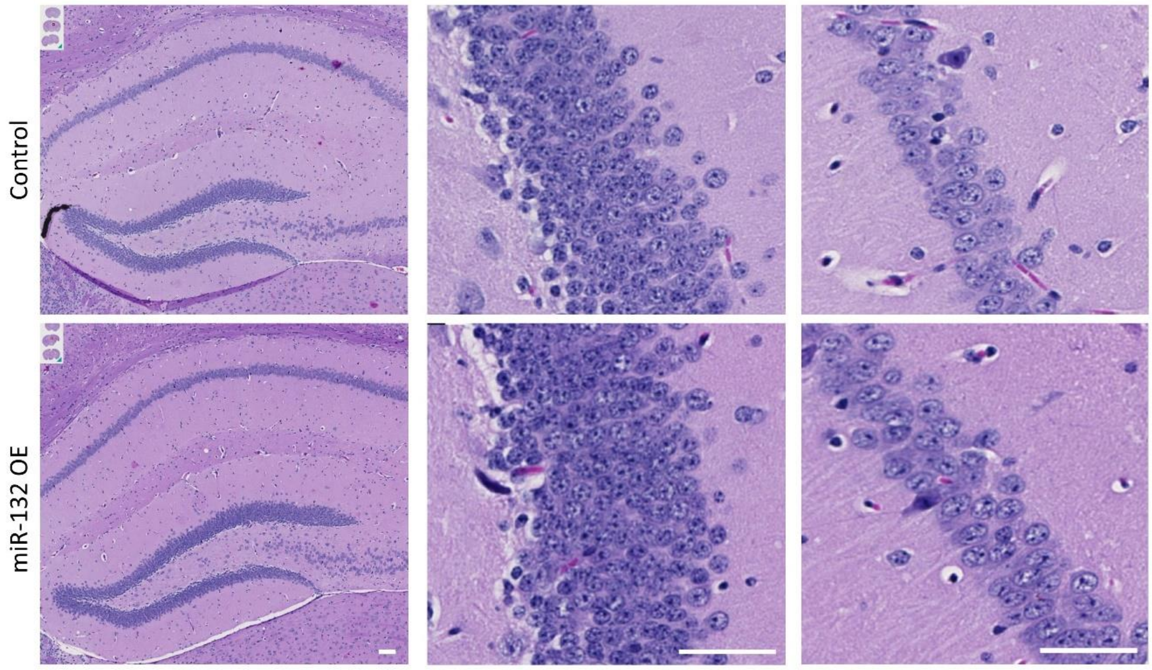

Supplementary Figure 4. Histopathological assessment

Representative light microscopy images of the whole hippocampus and zoom in of the dentate gyrus and CA1 region in the brain of *App*$^{NL\text{-}G\text{-}F}$ mice upon miR-132 or control AAV injection, stained with hematoxylin and eosin. Scale bars, 40 µm.

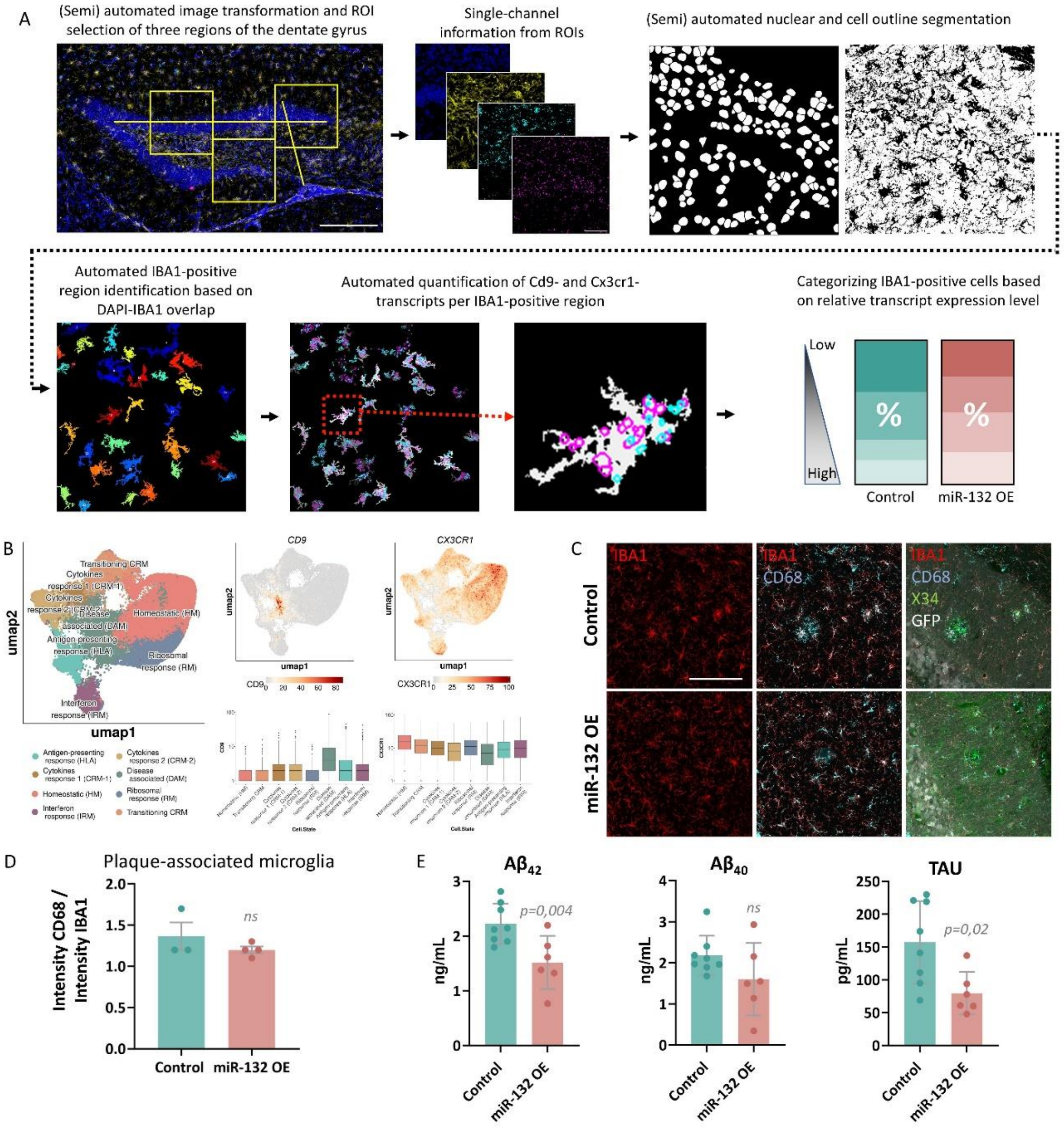

Supplementary Figure 5. Targeted miR-132 overexpression in AD mouse brain

(A) Overview of the analysis pipeline used to quantify transcript levels of *Cd9* and *Cx3cr1* in IBA^{+}-cells. (B) UMAPs and bar plots visualizing expression levels of *CD9* and *CX3CR1* using a single-cell RNA sequencing dataset derived from human iPSC-microglia xenotransplanted in *App*$^{NL\text{-}G\text{-}F}$ mice (Mancuso et al., Nat Neurosci, 2024). (C) Representative confocal images of the dentate gyrus in the brain of *App*$^{NL\text{-}G\text{-}F}$ mice, immunolabeled for IBA1 (red), CD68 (blue) and X34 (green) after miR-132 or control AAV injection (GFP, white). Scale bars, 100 µm. (D) Quantification of CD68 intensity normalized per microglial cell (IBA1). (E) Levels of TAU, $A\beta_{42}$ and $A\beta_{40}$ in the dentate gyrus of mice injected with either the miR-132 or the control AAV construct as measured by ELISA.

Supplementary Table 1. Information on iPSC donors

Diagnosis	iPSC Coriell #	Age at biopsy	Sex	APOE genotype	Reference	Symbol in figures
Healthy	AG28205	64	Male	E3/E3	Meyer 2019	▼
Healthy	AG27602	72	Male	E3/E3	Meyer 2019	★
Healthy	AG27611	75	Female	E3/E3	Meyer 2019	●
sAD	AG27605	72	Male	E3/E3	Meyer 2019	▼
sAD	AG27607	69	Female	E2/E3	Meyer 2019	●

Supplementary Table 2. Differential expression analysis in iMGs
Differential expression analysis was performed between miR-132 knockdown (KD) and control (ctr) group in healthy iMGs, between AD iMGs and healthy iMGs, between miR-132 overexpression (OE) and control group in AD iMGs, and between miR-132 overexpression and control group in healthy iMGs. logFC, average of log fold change; adj.P.Val, adjusted p-value using the Benjamini-Hochberg correction.

Supplementary Table 3. Gene ontology (GO) enrichment analysis
GO terms identified as significantly enriched (p-value < 0,05) when analysing DEGs either upregulated or downregulation between miR-132 knockdown (KD) and control (ctr) group in healthy iMGs, between AD iMGs and healthy iMGs, between miR-132 overexpression (OE) and control group in AD iMGs, and between miR-132 overexpression and control group in healthy iMGs.

Supplementary Table 4. Reference microglial gene lists
Lists of microglial cell state markers identified from previously published single-cell and single-nucleus RNA sequencing datasets of microglia, and list of AD risk genes used for Gene Set and Cluster Enrichment Analysis. The 'GSEA' sheet contains gene lists used for the analysis presented in Figure 2F. The 'Cluster enrichment analysis' sheet contains gene lists used for the analysis presented in Supplementary Figure 3D.

Supplementary Table 5. *In silico* identification of miR-132 targets
List of 732 predicted miR-132 targets derived from three different miRNA target prediction algorithms (TarBase, miRDB, miRDIP).

Supplementary Table 6. Putative direct miR-132 microglial targets
Complete lists of the miR-132 targetomes identified in all datasets and their overlap, as described in Figure 5. OE, overexpression; KD, knockdown.

Supplementary Table 7. Gene network analysis
Results of gene network analysis of putative direct miR-132 microglial targets overlapping between datasets. OE, overexpression; KD, knockdown.

Supplementary Table 8. Primers used

Primer	Forward	Reverse
hsa-miR-132-3p	UAACAGUCUACAGCCAUGGUCG	
mGapdh	TTGATGGCAACAATCTCCAC	CGTCCCGTAGACAAAATGGT
mActin	AGCCATGTACGTAGCCATCC	CTCTCAGCTGTGGTGGTGAA
mMapt	CGCCCCTAGTGGATGAGAGA	GCTTCTTCGGCTGTAATTCCTT
hRPLP0	CCTCGTGGAAGTGACATCGT	CTGTCTTCCCTGGGCATCAC
hUBC	GGGTCGCAGTTCTTGTTTGT	GGAGGGATGCCTTCCTTATC
hMAP2	GACTGCAGCTCTGCCTTTAG	AAGTAAATCTTCCTCCACTGTGAC
hGFAP	GAGGTTGAGAGGGACAATCTGG	GTGGCTTCATCTGCTTCCTGTC
hOLIG2	GACAAGCTAGGAGGCAGTGG	CGGCTCTGTCATTTGCTTCT
hCD164	AAGTGGGGAACACGACAGAC	TGAAACTGGCTGCATCAAAG
hIL-6	AGACAGCCACTCACCTCTTCAG	TTCTGCCAGTGCCTCTTTGCTG
hTNF-α	CTCTTCTGCCTGCTGCACTTTG	ATGGGCTACAGGCTTGTCACTC
hIL-1β	CCACAGACCTTCCAGGAGAATG	GTGCAGTTCAGTGATCGTACAGG
hOCT4	TCGAGAACCGAGTGAGAGG	GAACCACACTCGGACCACA
hMERTK	AGCGGGAGATCGAGGAGT	CCCCGTATTTCATGAAGGGTA
hHEXB	GGGAGCATTACGAGGTTTAGAG	GGTGGATTCATTGATGGTGAAAG
hPROS1	AAGAAGCCAGGGAGGTCTTTG	ACGTGCAGCAGTGAATAACC

Supplementary Table 9. Antibodies used

Antibody	Dilution	Source	Identifier
Rabbit anti-OCT4	1:200	Abcam	Ab19857
Mouse anti-TRA-1-81	1:300	Invitrogen	MA1-024
Mouse anti-TMEM119	1:100	ProteinTech	66948-1-Ig
Rabbit anti-IBA1	1:200	WAKO	019-19741
Rat anti-CD68	1:250	Abcam	Ab53444
Goat anti-mouse 568	1:500	Invitrogen	A11004
Goat anti-rabbit 488	1:500	Dylight	11-487-003
Goat anti-rat 647	1:500	Invitrogen	A21247
Donkey anti-rabbit 594	1:500	Jackson	711-585-152
